# Just-in-Time Adaptive Interventions for Behavior Change in Physiological Health Outcomes and the Use Case for Knee Osteoarthritis: Systematic Review

**DOI:** 10.2196/54119

**Published:** 2024-09-27

**Authors:** Janis Fiedler, Matteo Reiner Bergmann, Stefan Sell, Alexander Woll, Bernd J Stetter

**Affiliations:** 1 Institute of Sports and Sports Science Karlsruhe Institute of Technology Karlsruhe Germany

**Keywords:** eHealth, application, applications, digital health, digital technology, digital intervention, digital interventions, physical activity, sedentary behavior, sedentary lifestyle, pain, physical function, quality of life, knee osteoarthritis, osteopathy, bone, arthritis, mobile phone, individualization

## Abstract

**Background:**

The prevalence of knee osteoarthritis (KOA) in the adult population is high and patients profit from individualized therapy approaches. Just-in-time adaptive interventions (JITAIs) are upcoming digital interventions for behavior change.

**Objective:**

This systematic review summarizes the features and effectiveness of existing JITAIs regarding important physiological health outcomes and derives the most promising features for the use case of KOA.

**Methods:**

The electronic databases PubMed, Web of Science, Scopus, and EBSCO were searched using keywords related to JITAIs, physical activity (PA), sedentary behavior (SB), physical function, quality of life, pain, and stiffness. JITAIs for adults that focused on the effectiveness of at least 1 of the selected outcomes were included and synthesized qualitatively. Study quality was assessed with the Quality Assessment Tool Effective Public Health Practice Project.

**Results:**

A total of 45 studies with mainly weak overall quality were included in this review. The studies were mostly focused on PA and SB and no study examined stiffness. The design of JITAIs varied, with a frequency of decision points from a minute to a day, device-based measured and self-reported tailoring variables, intervention options including audible or vibration prompts and tailored feedback, and decision rules from simple if-then conditions based on 1 variable to more complex algorithms including contextual variables.

**Conclusions:**

The use of frequent decision points, device-based measured tailoring variables accompanied by user input, intervention options tailored to user preferences, and simple decision rules showed the most promising results in previous studies. This can be transferred to a JITAI for the use case of KOA by using target variables that include breaks in SB and an optimum of PA considering individual knee load for the health benefits of patients.

## Introduction

### Background

Osteoarthritis is one of the most common chronic joint conditions and leads to pain, stiffness, and reduced physical function, which in turn diminishes quality of life (QoL) [1,2]. The most commonly affected weight-bearing joints are the knees, followed by the hips [3]. In 2019, about 344 million people worldwide were living with an osteoarthritis severity level that could benefit from effective therapy, and this number will grow over the next few years [4]. Appropriate mechanical stimuli, including physical activity (PA), are joint health protecting factors [5]. Furthermore, an adequate level of PA and reduced sedentary behavior (SB) results in reduced pain and improves physical function and stiffness in patients with knee osteoarthritis (KOA) [6-10]. Thereby, the QoL of people that are affected can also be improved [11]. Treating pain and overcoming functional limitations are essential for preventing a downward spiral of reduced PA and declining QoL [12]. Inadequate PA can lead to excess bodyweight, which increases mechanical joint loading, for example, during locomotion, and accelerates the degenerative process of KOA [13]. Furthermore, individuals with KOA are more prone to cardiovascular disease, as factors, such as reduced PA, are also correlated with elevated cardiovascular disease risk [14]. Consequently, PA and exercise are recommended for preventing such disease [14]. Additionally, PA and exercise have been shown to be beneficial in terms of alleviating KOA symptoms such as pain and stiffness [15]. Previous research has highlighted the importance of new strategies to encourage patients with KOA to participate in greater levels of PA [13,16]. However, KOA is a highly heterogeneous and multifactorial disease that affects individuals differently with variations in disease progression, severity, and response to treatments [13]. Consequently, an individualized therapy approach is suggested to effectively manage the symptoms, slow down the progression of the disease, and promote a healthy lifestyle specifically tailored to each person [17]. While this is also possible for interventions that are delivered in person, mobile health (mHealth) interventions are upcoming methods with the opportunity to deliver such highly individualized interventions at a larger scale. mHealth interventions are defined as medical and public health practices supported by mobile devices, such as mobile phones, patient monitoring devices, PDAs, and other wireless devices [18]. These interventions profit from the fast development of accompanying sensors such as accelerometers and heart rate monitors [19], which allow for ecologically valid real-time interventions. Previous studies show promising results for PA promotion and the reduction of SB if a theoretical foundation and behavior change techniques (BCTs) were used for the intervention [20]. In the context of KOA, reviews point to good usability and the potential for effective interventions, but the research is in an early stage [21,22]. A review including mHealth interventions for KOA points out that most available apps lack scientific studies backing their effectiveness, but those evaluated by RCTs show promising results concerning pain, physical function, and PA [23]. The main features of these apps included exercise prescription and the tracking of symptoms. A special case of mHealth interventions of which patients with KOA could greatly benefit are the highly individualized just-in-time adaptive interventions (JITAIs) or ecological momentary interventions (from here on only the term JITAI is used). These aim to generate real-time or near–real-time feedback by leveraging prior knowledge of individuals and previously collected data. With this compiled, dynamic knowledge, tailored interventions and nudges can be delivered when needed through mobile technologies, aligned with the individual’s context and requirements [24]. These nudges can then change proximal outcomes such as step count to achieve the ultimate distal goals of the JITAI, such as better joint health and QoL [25]. An intervention is defined as a JITAI if it corresponds to real-time needs, adapts to input data, and is triggered by the system with the aim to deliver the intervention at a state of vulnerability, opportunity and receptivity of participants [25-28]. In the context of designing JITAIs, decision points represent the specific points in time when the JITAI can be triggered, while decision rules are the criteria that are applied at a decision point to determine if the JITAI should be triggered and which intervention option should be used. Intervention options refer to the possible actions that the JITAI can take at a decision point, while tailoring variables denote the sensor or user input that is used for adaptation [26]. A previous review on JITAIs for PA promotion by Hardeman et al [28] and a meta-analysis for device-based measured health outcomes by Xu and Smit [29] point to the feasibility and preliminary effectivity of JITAIs to promote PA and reduce SB. However, further variables (ie, physical function, stiffness, pain, and QoL) are important to KOA [8-11] that were not considered in previous works. Additionally, previous reviews did not aim to clearly depict decision points, tailoring variables, intervention options, and decision rules from the literature. This, however, is important to build a foundation for future JITAI designs. Finally, JITAIs are an upcoming field, with new studies getting published frequently warranting frequent updates of the literature.

### Objective

This review aimed to systematically summarize the literature regarding the features of JITAIs of important physiological health outcomes and proxies (ie, PA, SB, physical function, QoL, pain, and stiffness). The second aim was to extract the most promising features that should be considered by future JITAIs in the treatment of KOA.

## Methods

### Design

This systematic review was registered on the Open Science Framework [30] on March 7, 2022. It was carried out based on the PRISMA (Preferred Reporting Items for Systematic Reviews and Meta-Analyses) expanded checklist [31].

### Inclusion Criteria

Inclusion and exclusion criteria were formulated according to PICOS (Population, Intervention, Comparison, Outcomes, Study) and are presented in Textbox 1.

**Inclusion and exclusion criteria.**

1. Population: Participants in the studies must be aged >18 years, regardless of sex and gender. Thus, the only exclusion criterion relates to studies that only included participants aged <18 years of age. Studies were included if <50% of participants were aged >18 years.
2. Intervention: All studies that use a digital physiological treatment that can be defined as a JITAI are included in this review. If studies use other types of interventions in addition to JITAIs, they are still included, and only the JITAI parts were considered. Furthermore, there were no restrictions regarding the duration of the intervention, the number of intervention sessions, or the technical device for the intervention.
3. Comparator: There was no restriction regarding control. We included active control (eg, other physiological treatments or interventions), passive control (no intervention or treatment), and studies with no control group (CG).
4. Outcome: Studies were included whenever they evaluated the effectiveness of a JITAI regarding one of the parameters PA, SB, pain, QoL, physical function, or stiffness as a main outcome. This also included all parameters that are synonyms or components of the previously mentioned parameters.
5. Study design: We included all study types, except reviews or meta-analysis. The only important thing was that the study reported on the effectiveness of the intervention.

### Search Strategy

The electronic databases PubMed, Web of Science, Scopus, and EBSCO were used to identify relevant studies. Because search strategies exclusively relying on databases have been shown to be nonexhaustive [32], the reference list of all selected articles was checked for further relevant studies. Initial searches were conducted on October 26, 2022, and the search was rerun on April 9, 2024. We combined different search terms related to the intervention type, the area of mHealth, and the outcome parameters. Further details are available in Multimedia Appendix 1.

### Paper Selection

All identified studies were exported to a reference management tool (EndNote [Clarivate] or Citavi [Swiss Academic Software GmbH]). The reference management tools were used to remove all duplicates. Then, all titles were checked for eligibility by MRB and JF independently. Second, the abstracts were screened, and third, the full texts of all remaining papers were screened, and the reasons for exclusion were noted by the 2 authors. Any disagreements at one of the screening steps were resolved by discussion until consensus was found.

### Study Quality Assessment

The Quality Assessment Tool Effective Public Health Practice Project was used for the evaluation of the study quality. It is a specifically designed tool to test and support findings in public health research and refers to articles related to a broad number of health-related topics, including chronic diseases [33]. By evaluating 6 different criteria, a global rating was assessed at the end for each study. Every criterion could be rated *weak* (red), *moderate* (yellow), or *strong* (green), which finally influences the global rating. One *weak* rating leads to a *moderate* global rating, and 2 or more *weak* ratings result in a *weak* global rating. Only studies without a *weak* rating can achieve a *strong* global rating [33].

### Data Extraction

All important information of the selected studies has been reproduced in tabular form in the Results section. The preparation of the tables and their contents were developed by MRB and JF in consultation with BS. For the most accurate rendering of the study content, reference was made to the study characteristics, including study design, sample size, population, and setting, as well as characteristics of the participants. To provide starting points for future JITAIs, the features and delivery of the JITAIs, including decision points, tailoring variables, intervention options, and decision rules based on the JITAI framework [27], and the duration of the interventions were also extracted from the existing studies. To display effectiveness and theoretical foundation, all data regarding retention, measurement, and the significant within- or between-group differences of the intervention and the use of BCTs stratified by control and intervention group (IG) according to Michie et al [34], and the theoretical foundation of the interventions are displayed in the Results section.

## Results

### Study Selection

**Figure 1:**
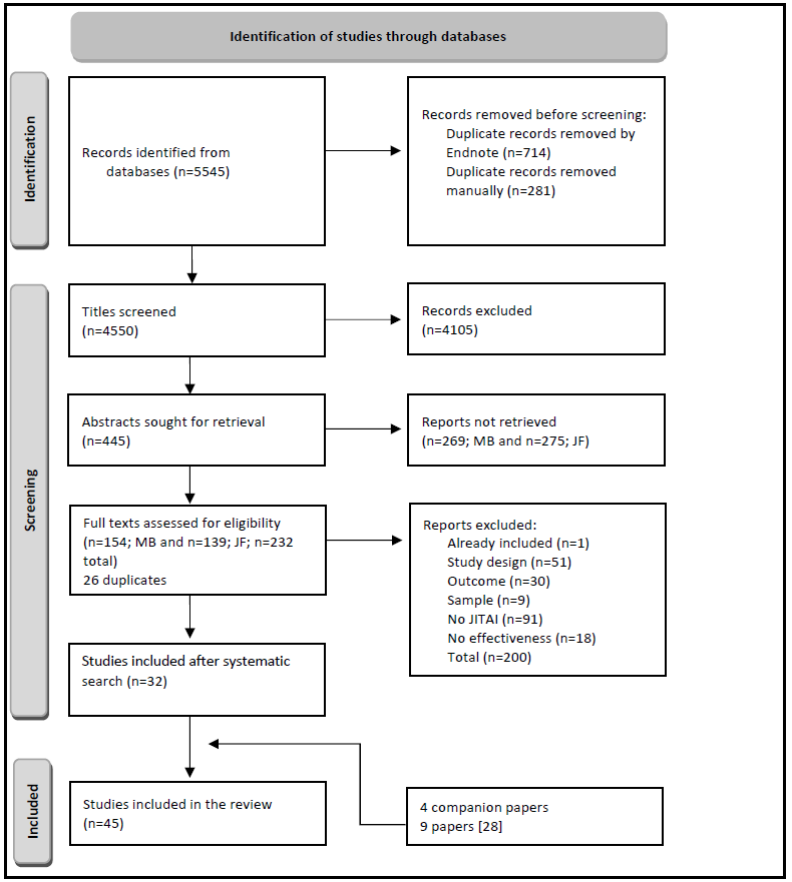
PRISMA (Preferred Reporting Items for Systematic Reviews and Meta-Analyses) flow diagram. JITAI: just-in-time adaptive intervention.

A total of 4340 papers were exported to Endnote (MRB) and Citavi (JF) and screened (Figure 1). Initially, 571 (13.16%) duplicates were excluded by the reference management tools, and another 279 (6.43%) duplicates were removed manually. The remaining 3490 (80.41%) papers were screened for their titles, and 3133 (72.19%) were excluded by the authors. Thus, 357 (8.23%) abstracts were read, and 213 (4.91%) more papers were excluded at this step. The updated search yielded 1215 (28%) additional papers, including 145 (3.34%) duplicates. The 1070 (24.65%) unique papers were screened for title and abstract, and the remaining 88 (2.03%) papers for full texts. The main reasons for exclusion were that it was no intervention study, the intervention did not qualify as a JITAI, the outcome did not fit the inclusion criteria, it was a systematic review or meta-analysis, or all participants were <18 years old. Full texts of the remaining 144 (3.32%) studies were screened, and finally 18 (0.41%) papers were included in the review, supplemented by additional 14 (0.32%) papers from the updated search. During the screening process, 13 more studies were added. Nine studies were adopted from the systematic review by Hardeman et al [28], and 4 additional papers were found during data extraction by checking references of the included studies. Finally, 45 studies are presented and summarized in Table 1 and Multimedia Appendices 2 [30-74] and 3 [35-79].

**Table 1:** Study characteristics.

Author and country of study	Study design	Population and activity profile	Analyzed sample size	Participant characteristics (age, gender, and ethnicity)
Allicock et al [36], United States	Feasibility study or pilot evaluation Randomized controlled trial	Female African American breast cancer survivors. Healthy enough to engage in moderate to vigorous-intensity PA^a^.	N=22 (IG^b^=13, 59%; and CG^c^=9, 41%)	Age: mean (SD)=52.2 (9.2) years No male participants. 100% (22/22) African American.
Baumann et al [37], Germany	Multiarm, parallel-group, randomized controlled trial	Health care professionals (nursing staff and office workers) aged 18 years and older	N=170 (IG 1=21, 12.4%; IG 2=23, 13.5%; IG 3=7, 4.1%; IG 4=34, 20%; IG 5=16, 9.4%; and CG=69; 40.6%)	Age: mean (SD)=41.1 (10.9) years. 39% (66/170) male participants
Bond et al [38], and Thomas and Bond [39], United States	Feasibility study or pilot evaluation Randomized within-person study The participants were randomized each week to one of the 3 different conditions	Adults who are overweight or obese No exclusion based on PA level	N=30	Age: mean (SD)=47.5 (13.5) years. 17% (5/30) male participants. 67% (20/30) White, 13% (4/30) African American, 3% (1/30) American Indian or Alaskan Native, 3% (1/30) Asian, and 13% (4/30) other
Bort-Roig et al [40], Spain	Randomized controlled trial	Desk-based employees at-work and away from work Low activity level outside work No physical or health problems that limited their ability to stand for bouts of at least 10 minutes	N=141 (IG=90, 63.8%; and CG=51, 36.2%) Follow-up: N=64 (IG=42, 66%; and CG=22, 34%)	Age: mean (SD)=45 (9) years 18% (25/141) male participants
Brakenridge et al [41,42], Australia	Cluster-randomized workplace trial Mixed methods evaluation	Desk-based office employees Not reported	N=153 (IG=66, 43.1%; and CG=87, 56.9%) Only IG data were used for 2018 results.	IG age: mean (SD)=37.6 (7.8) years 47% (31/66) male participants CG age: mean (SD)=40 (8) years. 60% (52/87) male participants
Carlozzi et al [43], United States and Wang et al [44], United States	Carlozzi et al: feasibility study or pilot evaluation Randomized controlled trial. Wang et al: pilot evaluation. 2-arm microrandomized controlled trial.	Care partners who are aged 18 years and are caring for an individual aged 18 years or older.	Carlozzi et al N=70 (IG=36; 51%; and CG=34, 49%) Wang et al N=36 (IG of Carlozzi et al [43])	IG age: mean (SD)=54.4 (13.1) years 22% (8/36) male participants 81% (29/36) Caucasian, 3% (1/36) African American, and 8% (3/36) Asian CG age: mean (SD)=56.1 (14.5) years 35% (12/34) male participants. 97% (33/34) Caucasian and 3% (1/34) African American
Compernolle et al [45], Belgium	Single-group pre-post design	Age >60 years Able to walk 100 m without difficulties	N=26	Age: mean (SD)=64.4 (3.8) years 50% (13/26) male participants 100% (26/26) Flemish
Conroy et al [46], United States	Pilot evaluation Secondary data analysis using a within-person design	Insufficiently active young adults aged between 18 and 29 years	N=58	Age: mean (SD)=24.6 (3.1) years 31% (18/58) male participants. 69% (40/58) White and 31% (18/58) other
Ding et al [47], United States	Feasibility study or pilot evaluation Randomized controlled trial	College students. Participants who were in the contemplation and preparation stage based on the transtheoretical model.	N=16 (IG=9, 56%; and CG=7, 44%)	Age: 18-25 years 63% (10/16) male participants. Other characteristics were not reported.
Direito et al [48], New Zealand	Feasibility study or pilot evaluation Single-group pre-post design	Adults who did not meet PA recommendations or who did meet recommendations but intended to decrease sedentary time.	N=69	Age: mean (SD)=34.5 (11.8) years 22% (15/69) male participants 54% (37/69) New Zealand European, and 46% (32/69) other
Fiedler et al [49], Germany	Cluster-randomized controlled trial Secondary data analysis using a within-person design for the IG	Families (at least 1 parent and 1 child) Not reported	N=80	59% (47/80) adults 53% (42/80) male participants
Finkelstein et al [50], United States	Feasibility study or pilot evaluation Randomized controlled crossover study	Sedentary, overweight women Daily inactivity >3 h	N=30	Age: mean (SD)=52 (12) years No male participants 47% (14/30) White and 47% (14/30) African American
Freene et al [51], Australia	Feasibility study or pilot evaluation Single-group pre-post design	Adults with a stable coronary heart disease Able to perform a submaximal walking test	N=20	Age: mean (SD)=54 (13) years. 85% (17/20) male participants 61% (12/20) Australian
Fundoiano-Hershcovitz et al [52], Israel	Retrospective cohort study	Adults with high pain levels and >6 weekly training hours (users with <6 weekly training hours were used for the sensitivity analysis).	N=981	Age: mean (SD)=39.2 (29) years 39% (383/981) male participants
Garland et al [53], United States	Feasibility study or pilot evaluation Randomized controlled trial	Adult patients who are opioid-treated for chronic pain	N=63	Age: mean (SD)=53.6 (12.8) years 59% (37/63) male participants 92% (58/63) White and 8% (5/63) other
Golbus et al [54], United States	Randomized clinical trial	Low to moderate risk patients aged between 18 and 74 years in cardiac rehabilitation	N=223 (IG=112, 50.2%; and CG=111, 49.8%)	Age: mean (SD)=59.6 (10.6) years 69% (154/223) male participants 84% (187/223) White and 16% (36/223) others
Hermens et al [55], and Tabak [56], the Netherlands	Feasibility study or pilot evaluation Quasi-experimental study Single-group pre-post design	People living with COPD^d^ who had completed a lung rehabilitation program 3 mo before the study	N=8	Aged between 49 and 64 years 60% (5/8) male participants
Hietbrink et al [57], the Netherlands	Mixed methods longitudinal study	Adults with type 2 diabetes	N=20 N=15 (PA module) N=5 (nutritional module)	Age: mean (SD)=68 (8) years. 70% (14/20) male participants
Hiremath et al [58], United States	Nonrandomized pilot evaluation Single-group pre-post design	Individuals with SCI^e^ Use a manual wheelchair and self-propel them No active pelvic or thigh wounds, CVD^f^, or pregnancy	N=20	Age: mean (SD)=39.4 (12.8) years 80% (16/20) male participants 12.4 (12.5) years since injury
Ismail and Al Thani [59], Qatar	Experimental study (between-group design) Controlled trial	Adults with a predominantly sedentary job Able to walk	N=58 (IG=29; 50%; and CG=29, 50%)	Aged between 23 and 39 years 34% (10/29) male participants (IG) and 41% (12/29) male participants (CG)
Klasnja et al [60], United States	Feasibility study or pilot evaluation Single-group pre-post design 2×2 factorial experiment Within-person microrandomized trial	Adults who are obese after bariatric surgery	N=45	Age: mean (SD)=45 (11.9) years 16% (7/45) male participants 80% (36/45) White, 7% (3/45) Black, 2% (1/45) other, and 7% (3/45) mixed
Li et al [61], United States	Feasibility study or pilot evaluation Single-group pre-post design	No prior diagnosis of cognitive impairment or dementia, sedentary lifestyle (self-reported >6 hours of sitting activities per day), poor sleep quality (insomnia severity index ≥8), and no diagnosis of sleep apnea	N=8	Age: mean (SD)=74 (5.42) years 25% (2/8) male participants 50% (4/8) White and 50% (4/8) Black
Low et al [62], United States	Usability and feasibility study Single-group pre-post design	Adults with abdominal cancer postsurgery Able to stand and walk unassisted	N=15	Age: mean=49.7 years 20% (3/15) male participants 87% (13/15) White and 13% (2/15) Black
Low et al [63], United States	Feasibility study or pilot evaluation Randomized controlled trial	Adults scheduled for surgical treatment of metastatic gastrointestinal or peritoneal cancer and able to stand and walk unassisted	N=26 (IG=13, 50%; and CG=13; 50%)	Age: mean (SD)=56.2 (10.5) years 58% (15/26) male participants 92% (24/26) White, 4% (1/26) Black, and 4% (1/26) more than one
Martin et al [64], United States	Randomized clinical trial	Outpatients of an academic cardiac vascular disease prevention center Self-reported moderate or vigorous leisure-time ≥ 30 min/day at <3 days/week	N=48 (unblind, no texts=16, 33%; Unblind, texts=16, 33%; and blind=16, 33%).	Age: mean (SD)=58 (8) years. 54% (26/48) male participants 79% (38/48) White
McEntee et al [65], United States	Randomized trial	Adults who are inactive and healthy	N=512 (n=128, 25% per study group)	Age: mean (SD)=45.5 (9.1) years 36% (184/512) male participants 19% (97/512) Hispanic, 6% (31/512) African American, 2% (10/512) Asian, and 84% (430/512) White
Nurmi et al [66], Finland	Pilot evaluation N-of-1 randomized controlled trial	General population >18 years No contraindications to engaging in PA, no use of any PA trackers or PA apps in the previous 6 months, no participation in other trials or behavior change programs in the previous 6 months or during the trial, below 150 MVPA^g^/week	N=15	Age: mean (SD)=42.3 (9.8) years 27% (4/15) male participants
Pellegrini et al [67], United States	Feasibility study or pilot evaluation Quasi-experimental study Single-group prepost design	People living with type 2 diabetes Sedentary occupation or spend ≥75% of the day sitting	N=9	Age: mean (SD)=53.1 (10.7) years 23% (2/9) male participants 23% (2/9) White and 77% (7/9) Black
Rabbi et al [68], United States	Feasibility study or pilot evaluation, within-subject Randomized controlled trial	Volunteers, including students and professionals Not reported	N=17	Age: mean (SD)=28.3 (6.96) years, 18-49 years 53% (9/17) male participants
Rabbi et al [69], United States	Feasibility study or pilot evaluation Quasi-experimental study Controlled within-person trial	Employees of Cornell University Not reported	N=16	Age: 18-29 years: 25% (4/16), 30-39 years: 38% (6/16), 40-49 years: 19% (3/16), and >50 years: 19% (3/16). 44% (7/16) male participants
Rabbi et al [70], United States	Feasibility study or pilot evaluation Single-blinded, controlled within-person trial	Adults with chronic back pain (>6 months in duration) Able to walk without mobility aids	N=10	Age: 31-60 years 30% (3/10) male participants
Radhakrishnan et al [71], United States	Feasibility study or pilot evaluation Randomized controlled trial	Adults with heart failure Able to walk	N=38 (IG=19; 50%; and CG=19; 50%)	Age: 55 years or older 53% (20/38) male participants 76% (29/38) White, 16% (6/38) African American, 3% (1/38) Native American, and 5% (2/38) other
Robertson et al [35], United States	Feasibility study or pilot evaluation Randomized controlled trial	Adult cancer survivors Did not meet the recommended activity levels	N=78 (IG=39, 50%; and CG=39; 50%)	Age: 18-34 years: 5% (4/78), 35-49 years: 26% (20/78), 50-64 years: 33% (26/78), 65-74 years: 33% (26/78), and >74 years: 3% (2/78) 9% (7/78) male participants 1% (1/78) American Indian or Alaska native, 17% (13/78) Black or African American, 79% (62/78) White, and 1% (1/78) other
Sporrel et al [72], the Netherlands	Feasibility study or pilot evaluation Randomized controlled trial design with single-group pre-post evaluation	Adults between 18 and 55 years Did not meet WHO^h^ PA guidelines and no medical condition that made it unsafe to engage in unsupervised PA	N=20 (Smart PAUL^i^=11, 55%; and Basic PAUL=9, 45%)	Age: mean (SD)=30.65 (8.4) years 15% (3/20) male participants
Stuber et al [73], the Netherlands	Parallel cluster-randomized controlled trial	30 to 80 y old adults, regular shoppers at a participating supermarket (self-reported to purchase >50% weekly groceries at a participating supermarket)	N=361 (IG=162, 44.9%; and CG=199, 55.1%)	IG age: mean (SD)=58.9 (11.5) years 29% (105/361) male participants CG age: mean (SD)=57.2 (10.2) years 26% (94/361) male participants
Tabak et al [74,75], the Netherlands	Randomized controlled pilot trial Secondary data analysis using a within-person design for the IG	Patients with COPD A clinical diagnosis of COPD, no infection or exacerbation in the 4 wk prior to start of the study, and a current or former smoker	N=30 (IG=14, 47%; and CG=16, 53%)	IG age: mean (SD)=65.2 (9) years 57% (8/14) male participants. CG age: mean (SD)=67.9 (5.7) years 69% (11/16) male participants
Valle et al [76,77], United States	Randomized controlled trial	18 to 39 years old patients with a cancer diagnosis in the previous 10 years engaging in <150 min/week of MVPA	N=280 (IG=140, 50%; and CG=140; 50%)	Age: mean (SD)=33.4 (4.8) years 18% (50/280) male participants 23% (64/280) racial and ethnic minorities
van Dantzig et al [78], the Netherlands	Feasibility study or pilot evaluation Study 1: qualitative study Study 2: randomized controlled trial	Study 1: office workers Study 2: healthy office workers Study 1: not reported Study 2: sedentary job, no known physical handicap or other condition that makes PA (walking) impossible, older than 30 years, not participating in another PA intervention	Study 1: N=8 Study 2: N=86 (IG=40, 47%; and CG=46, 53%)	Study 1 age: not reported 50% (4/8) male participants Study 2 IG age: mean (SD)=44.5 (7.9); 30-57 years 58% (23/40) male participants. CG age: mean (SD)=44.3 (8); 32-63 years 63% (29/46) male participants
van Dantzig et al [79], the Netherlands	Substantive evaluation Randomized controlled trial	Desk workers with regular (daytime) working hours Not reported	N=70 IG and CG not reported	Age: 18-65 years 73% (51/70) male participants (based on N=70 recruited)

^a^PA: physical activity.

^b^IG: intervention group.

^c^CG: control group.

^d^COPD: chronic obstructive pulmonary disease.

^e^SCI: individuals with spinal cord injury.

^f^CVD: cardiovascular disease.

^g^MVPA: moderate to vigorous physical activity.

^h^WHO: World Health Organization.

^i^PAUL: The Playful Active Urban Living app.

### Description of the Included Studies

A total of 25 (56%) studies were conducted in the United States, 9 (20%) in the Netherlands, 2 (4%) in Australia, 2 (4%) in Germany, and the others in New Zealand, China, Qatar, Finland, Israel, Spain, and Belgium. Most of them (26/45, 58%) were designed as feasibility study or pilot evaluations, and they were mainly randomized controlled trials (23/45, 51%). In total, 9 of 45 (20%) were conducted in a single-group pre-post design, 3 (7%) as a secondary analysis using a within-person design for the IG, 2 as a controlled within-person trial, 2 (4%) as a mixed-method evaluation, and all other studies were either a retrospective cohort study, a randomized within-person study, or combined different study designs. The minimum number of participants was 8 (3 studies), and the maximum was 981 participants with an average of 99 (SD 175) participants. Two studies focused exclusively on women, and overall, 62% (2434/3960) of all participants were women and 38% (1516/3950) were men. The mean age in 19 studies was >50 years, and they focused mainly on a specific population such as sedentary people, patients with chronic diseases (eg, chronic obstructive pulmonary disease and coronary heart disease), or survivors of cancer. Retention in 3 studies was <50%, 4 studies were below 80%, 21 were ≥80% and <100%, and the remaining 12 studies had full retention. PA was the most common outcome (39 studies), followed by 19 studies that used parameters of SB as an outcome, of which 18 studies considered both PA and SB. In total, 9 studies examined the impact on QoL, 4 used synonyms of physical function, and 3 studies used pain as an outcome. None of the included articles referred to stiffness. Detailed results are displayed in Multimedia Appendix 3. An average of 8 BCTs (SD 7) were included for the IG and 4 (SD 5) for the CG. The number of BCTs in the IG ranged from 2 to 47, compared with 1 to 27 in the CG. The intervention duration ranged from 1 day to a maximum of 12 months, with a typical duration of 3 to 6 weeks. One study had no time restriction but a step count of 166.000 as a cutoff for the intervention duration [35]. Details on BCTs and theoretical foundations can be found in Multimedia Appendix 4 [35-79].

### Key Features of JITAIs

Detailed key features of the included JITAIs are displayed in Multimedia Appendix 2.

Most decision points at which a JITAI could potentially be triggered were set to short time intervals, which were either specified to one decision point each minute (n=11) or not clearly specified but indicating short time intervals (n=21). Furthermore, 5 studies set decision points to time frames of 10 minutes up to 2 hours, and 11 were set to multiple times per day up to daily.

Tailoring variables used for JITAI adaptation were mainly device-based measured by accelerometer, smartphone, or smartwatch and concerned PA and SB outcomes supplemented with variables such as time, weather, or location. A total of 10 studies used self-reported tailoring variables through a baseline questionnaire [64], through ecological momentary assessment (EMA) [36,43,44,57], or through nonspecified methods to assess pain and fatigue [62,63], to enable nonspecified user input [53], or to supplement PA data if the device has not been worn [68-70].

Intervention options at decision points included mainly the delivery of an audible or vibration prompt to participants accompanied by tailored feedback, information about health behavior, and suggestions for behavioral alternatives. One study explicitly explained in the prompt why the prompt occurred [47]. The alternatives based on the decision rules were mainly not to send the prompt for the decision points with short time frames, while those with longer timeframes had different intervention options depending on the tailoring variables.

Decision rules defining opportune moments were mainly based on if-then conditions. This means that an opportune moment was defined if a certain threshold —for example, 20 minutes of SB [67], <100 steps in 1 hour [49], a bout (≥3 min) of moderate to vigorous physical activity (or higher) PA was performed [58], lumbar posture [41,42], time, location, or weather-based thresholds [78] were met. Most decision rules were based on one if-then condition, while some used if-then decision trees. More complex models applied multiarmed bandit algorithms (sequential decision-making algorithm [80]) [68-70], pareto-frontier algorithm (taking user preferences into account [81]) [69], prelearned reinforcement learning models (models that make use of historical data and of data during the trial [82]) [72], k-nearest neighbor classifier (one of the most fundamental and simple classification methods [83]), and real-time prediction and self-learning by a support vector machine (a classical machine learning technique [84]) [55,56]. If the conditions for an opportune moment were met, intervention options were delivered, which included both fixed responses and microrandomized suggestions (eg, Klasnja et al [60]) for behavior change.

### Effectiveness

Detailed results for intervention effectiveness are displayed in Multimedia Appendix 3. For RCTs, 5 studies indicated effectiveness compared with a CG for PA [35,37,64,65,68], 3 studies for SB [37,63,79], and 1 study for pain [53]. For pre-post–designed and within-person studies, 8 studies indicated effectiveness in most outcome variables of the study for PA [38,39,46,49,57,61,67,69], 5 for SB [38,39,52,61,67], 1 study for pain [52], and 1 study for QoL [51]. Regarding the opportune-moment identification, decision points, and rules, most interventions that indicated effectiveness had near real-time decision points on a minute-to-minute basis, and simple decision rules depicting opportune moments when a threshold of 20 to 120 minutes of inactivity, SB, or computer activity was reached. Of these, the studies of Rabbi et al [68,69] were the only ones that used more complex algorithms. Effectiveness was also found for longer decision point periods: 2 times per hour [35], 2 to 3 times per day [57,64], and daily [51]. Effective interventions had an intervention duration between 3 weeks and 12 months, where the majority of studies had an intervention length between 3 and 8 weeks. The other included studies that indicated no or limited effectiveness and reported heterogeneous features regarding decision points, decision rules, opportune-moment identifications, and the complexity of algorithms as well.

### Study Quality

**Figure 2:**
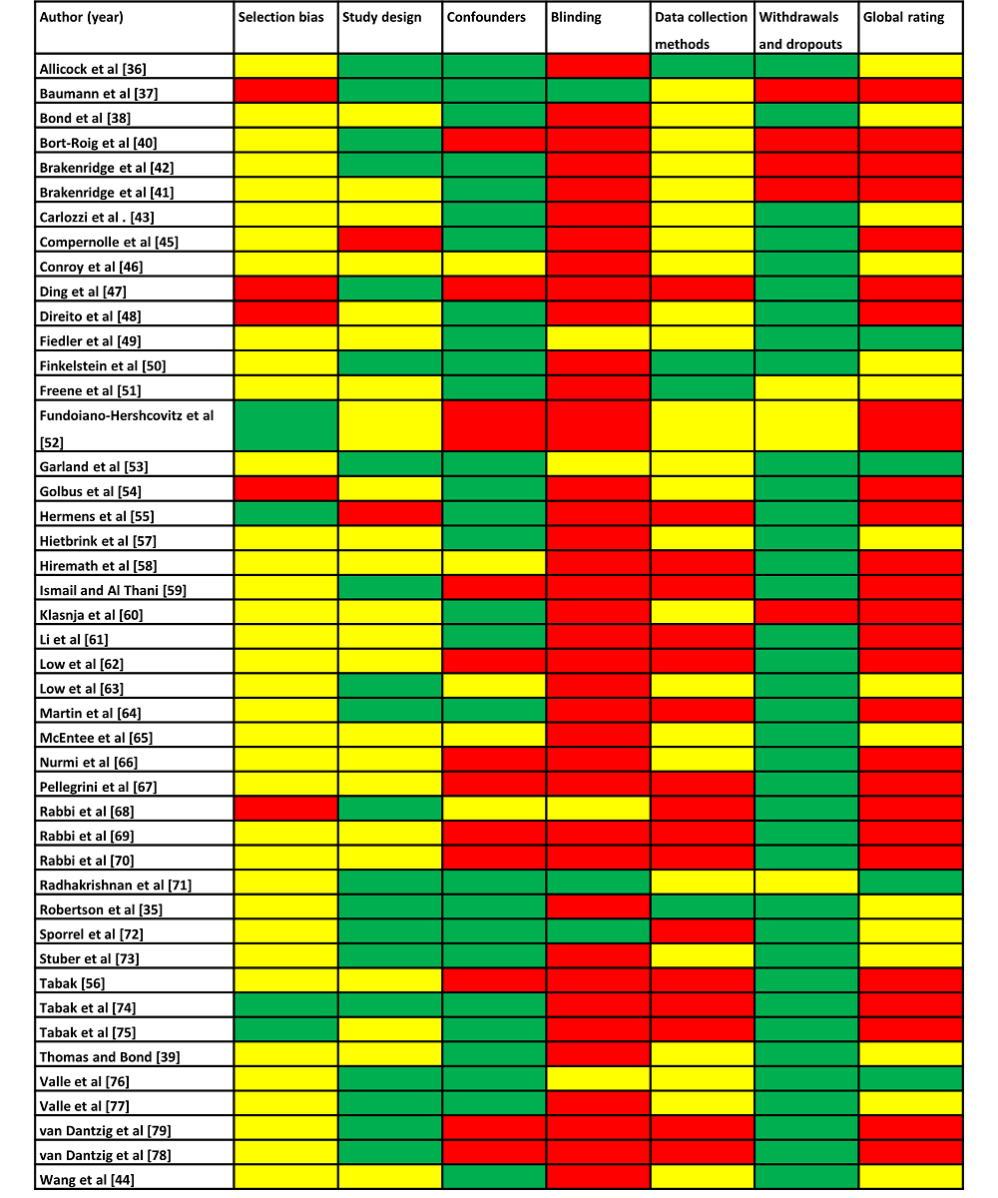
Study quality of included studies based on the Quality Assessment Tool Effective Public Health Practice Project. Green indicates a strong rating, yellow a moderate rating, and red a weak rating of the respective category.

The quality assessment showed that 4 studies achieved a “strong” global rating (Figure 2 [35-79]). Overall, 15 studies were rated “moderate,” and all the other studies were judged with 2 or more “weak” ratings. The most common weak ratings in 38 and 17 out of 45 were related to the context of blinding and data collection methods, respectively.

## Discussion

### Overview

The objective of this study was to provide a systematic overview of the concept of JITAIs and to explore important parameters in KOA management. In total, 45 studies with 38 different JITAIs focusing on the outcomes PA, SB, pain, QoL, physical function, and stiffness were analyzed in this review. The majority of studies investigated PA and SB, while physical function, pain, and QoL were addressed by few studies, and the parameter “stiffness” was not included in any study, indicating a research gap. Reporting and use of the key facets of JITAIs [27], along with the methodology and aspects of study design of the included studies, were heterogeneous. The best evidence for effective JITAIs exists for device-based measurements of PA and SB using frequent decision points and simple algorithms to deliver prompts after a period of physical inactivity or SB. This can be translated to the use case of KOA and accompanied by a specific knee load management to improve KOA treatments with JITAIs in the future.

### JITAIs in the Context of PA, SB, Pain, Stiffness, Physical Function, and QoL

Overall intervention effectiveness is hard to pinpoint and not comparable between studies due to heterogeneous study design, outcome measures, number of statistical comparisons, time windows, sample size, and focus on within- or between-person effects. Stratifying the results by study design, 5 out of 14 RCTs found the intervention to effectively increase PA and 3 studies had a positive effect on SB, and 1 study improved the pain level of participants. Pre-post study designs as well as the within-person studies found an increase in PA in 8 studies, a reduction of SB in 5 studies, a reduction of pain in 1 study, and 1 study positively influenced QoL. No evidence of effectiveness in studies for physical function outcomes was found. To obtain more results about causation under consideration of time-varying effects, it is important to increase the number of microrandomized trials in the future [60] and focus on the state of receptivity of participants. This will also provide more information about what works in which situation and for whom, and it will help to provide valuable information to advance the field of JITAI research.

Although the studies showed a high degree of heterogeneity, some patterns can be observed. For example, decision points with a shorter period (eg, minute-to-minute) and an intervention duration of 3 to 8 weeks have shown good evidence for intervention effectiveness. Here, it is important to add that intervention duration and timing of decision points have to be viewed together to avoid overburdening participants and to keep the content engaging. Otherwise, JITAIs run at a high risk of disengagement [85] that can hinder behavior change. The optimal dose for these parameters has yet to be completely explored. Conroy et al [46] point out that self-monitoring prompts, but not behavioral feedback, show signs of a dose-response association with PA volume in JITAIs. This is an important finding that should be followed up by further studies. The included studies provide limited information regarding the long-term effectivity of JITAIs for behavior change. Of the 15 studies that included a follow-up measurement, 3 studies showed significant benefits of the intervention for QoL [51,55,56] and 2 studies for PA [76,77]. This is not surprising, as JITAIs are an upcoming topic and still focus mainly on the opportune-moment identification, feasibility, usability, and technological challenges of the intervention [27]. In addition, decision rules that represent opportune moments when a threshold of 20 to 120 minutes of inactivity, SB, or computer activity has been reached resulted in behavior change. Here, shorter timeframes might be more useful to break SB, as Thomas and Bond [39] have shown that opportune moments after 30 minutes of being sedentary are more effective than longer decision points of 60 and 120 minutes.

PA or SB was monitored in all significant studies by the smartphone app or by an accelerometer. In addition, prompts were used by each study as an intervention option to interrupt SB, send encouraging messages, give suggestions to increase PA, or send useful and interesting links and information. For example, Freene et al [51] sent prompts in the form of do’s to help change behavior and learn a new behavior. The unique feature of this JITAI was that the do’s were sent based on a variety of tailoring variables, namely if participants showed a low score in PA, social opportunity, and variety on 3 consecutive days. Although it was not possible to positively influence PA or SB, the parameter QoL could be increased in 4 different domains, both after 6 and after 16 weeks. This result could mean that variety and social opportunity can be more easily influenced by do’s than PA. Another reason could be that shorter decision points are more effective for PA while for QoL longer decision points such as days are sufficient.

Importantly, Ding et al [47] highlighted that 43% of the supposed “just-in-time messages” were not perceived as just-in-time by the participants. This is problematic, as sending prompts at moments when participants do not have the opportunity to change their behavior will not lead to the desired effect and might even lead to disengagement with the intervention [26]. The importance of adequate opportune-moment identification has also been highlighted by previous research [25,27] and should be assessed by future JITAI studies. This can be done by a repeated short EMA, which then can also inform and adapt the timing of the intervention and improve adherence and thus behavior change. One example is the study of Rabbi et al [70], who included participants preferences into the opportune-moment identification, which allowed their algorithm to, for example, adapt learned opportune moments that were no longer valid.

Shortcomings of the included studies include the overall weak study quality and the mainly simple intervention design that is not optimized for opportune-moment identification as it seldomly includes the state of receptivity of participants but focuses on the vulnerability. This should be the focus of future studies that consider participants to, for example, not initiate a JITAI trigger when the tailoring variables (eg, calendar or user preferences) indicate that the participant is not receptive [86]. In this context, the role of contextual factors assessed by GPS or EMA [87] are important aspects that are included in a few studies [36,51,54-57,59,60,68-70,72,78,79] and point to the feasibility of such interventions. If interventions including context variables are more effective than those that do not, this cannot be answered by this review as no clear pattern emerges. The additional information provided by these studies is nonetheless very helpful for future interventions and should be explored further, including EMA studies on this topic [88]. The blinding of participants was the main issue for the quality assessment. While it is difficult to blind participants in mHealth research, microrandomization makes this process easier and has a lot of additional benefits, as discussed before. Furthermore, the included studies scored weak to moderate for selection bias. Here, more diverse samples and nonconvenient samples are required in future research to strengthen the evidence base and the generalization of JITAI study results. Additionally, future studies should test different sets of decision points, tailoring variables, decision rules, and intervention options within one study to improve the current literature on what works for whom and how. This would improve the evidence base for future JITAI interventions in different populations and settings.

### Specific Considerations for the Transfer of the Finding to the Use Case of KOA

As a recommendation, the World Health Organization [17] states that a healthy lifestyle should be cultivated and that maintaining a normal body weight plays an important role in preventing osteoarthritis and control the disease progression. Here, PA and SB are behavioral variables that influence pain, physical function, stiffness, and QoL of patients with KOA [6-10]. Consequently, PA and SB can be used to a certain extent as proxy variables to target the distal outcomes pain, physical function, stiffness, and QoL. However, even though appropriate mechanical stimuli are needed for promoting optimal joint function [5], more PA is not necessarily better for patients with KOA [89] and can even support the development of the disease [62]. It is recommended to reduce overuse of joints by refraining from PA that contain start-stop movements, rapid changes in direction, intense jumps, and landings [90].

To design a JITAI for KOA, specific tailoring variables have to be considered. On the basis of our findings, starting points for intervention studies could include device-based measured PA and SB variables in addition to time, location, and pain scores assessed as user input through a mix of event-based and random sampling using EMAs [68] to tailor the intervention to participants needs. From a theoretical perspective, additional JITAIs for stiffness would be very helpful to provide an evidence base for the creation of a JITAI for KOA. A further important tailoring variable that has not been found by this review is the knee load. Here, body-worn measurement technologies such as orthopedic shoe insoles or knee braces with built-in sensors to measure the forces exerted on the knee joint are promising developments for interventions that can adapt the minimum and maximum load limit to each participant and situation [91-94]. Wearable joint monitoring technologies may also help indirectly assess varying levels of joint stiffness.

For example, by quantifying the joint range of motion [95] or assessing relationships between gait biomechanics and fluctuations in KOA symptoms, valuable data can be gained [96]. Additionally, EMA could be used for a targeted assessment of specific symptoms, such as morning stiffness. Pain can also be assessed as user input through a mix of event-based and random sampling using EMAs [68]. This would allow to tailor the JITAI to, for example, a user specific self-reported amount of pain, or knee load under consideration of certain conditions and allow for a highly individualized approach.

A further important intervention method found by this review are educational prompts. These prompts could contain informative facts to educate the user on how to move and exercise appropriately [17] tailored to, for example, their PA, SB, pain, and stiffness. While conventional JITAIs mainly aim to increase PA through classical exercise, JITAIs that focus on the therapy of specific diseases, such as KOA, could use therapeutic exercises and tasks to promote health behavior. For example, it has already been shown that a combination of muscle strengthening and range-of-motion exercises resulted in a significant improvement in osteoarthritis symptoms in the long term [3,97]. Moreover, muscle strengthening was associated with reduced pain and increased function in patients with KOA [98]. These exercises could be encouraged in appropriate moments through the use of a JITAI. In the deployment of digital interventions and JITAIs, it is imperative to account for the technological prerequisites and potential barriers for participants and practitioners. This consideration becomes especially significant when the intended audience includes older patients, exemplified by those with KOA, as underscored in the research conducted by Hinman et al [23].

### Designing a JITAI for the Use Case of KOA

Several important decisions need to be made to design a JITAI for KOA. First, the outcome parameters need to be selected. Second, the device for JITAI delivery and assessment of tailoring variables needs to be chosen. Third, tailoring variables, decision points, intervention options, and decision rules need to be defined.

Derived from previous research and the results of this review, the aim of a promising JITAI is to target the proximal outcomes PA and SB in accordance with the recommendations of KOA therapy [17]. This should result in positive developments in the distal outcomes of pain, physical function, stiffness, and QoL. Here, smartphones, accelerometers, or KOA-specific measurement technologies can be used for the intervention and the evaluation of effectiveness. In addition to device-based measured PA and SB, EMAs can be used to gather information through user input about loads of previous activities or provide inferences about parameters such as pain and stiffness. The potential of EMAs for understanding individual behavior is indispensable [99,100] and 2 studies including EMA showed significant pain reduction in participants [52,53]. In addition, the use of sensors for load management would be very useful to provide device-based feedback of knee load and adapt the intervention accordingly to prevent an overloading of the knee joint [93,94]. Further approaches could also include smartphone data about the weather, calendar, or the user’s location to improve the opportune-moment identification [27].

The time span in which the decision points are to be made should be of shorter duration, as emerged from the results. Decision points every minute proved to be useful and offer quick adaptation to changing situations. That is especially important to avoid too high knee load in near real time and to break up SB. Prompts to boost overall PA and deliver knowledge could use longer decision points to avoid overburdening the participants [26].

The best evidence regarding intervention options exists for prompts to break prolonged SB or physical inactivity. That is also a relevant parameter for KOA [9] and could be linked with positive reinforcement messages or booster messages, which showed success in the study by Martin et al [64]. Additional intervention options include maintaining an optimal amount of PA while avoiding overloading of the osteoarthritis-affected knee. This could be achieved by continuously tracking and displaying the estimated knee load (eg, using knee-specific feedback technology [94,101]) on the smartphone and prompting participants in near real time if the knee load reaches a critical individual level. To avoid insufficient PA of participants, daily PA goals could be used. Here, daily prompts with positive reinforcement messages could prompt participants in the morning if they failed to achieve the PA goal of the previous day.

In the next step, the decision rules can be formulated. On the basis of the study by Thomas and Bond [39], interrupting SB after 30 minutes is promising, which is also linked to improved metabolic health [102]. Here, it is important to consider that the prompt really appears in an opportune moment [103] to avoid overburdening participants, especially if such a short time frame is chosen. One option to solve this is to allow users to choose time frames when such triggers occur, enable them to mute the triggers for a certain period, or use more complex decision-making algorithms based on machine learning principles that implement the availability of the user (eg, including calendar). Rules for accompanying positive reinforcement messages and educational information about why PA is useful in the topic of KOA should be formulated to have a maximum number of messages per day. There is also the possibility of including movement recommendations to go for a walk in the nearest park or to explore new places. Decision rules for the optimal amount of PA should only send prompts if participants are closing in on their critical knee load level or if inappropriate kinds of PA such as reoccurring jumps are detected to avoid making participants hesitant about PA. These prompts should always be followed by an explanation for the prompt and recommendations for the future. As these prompts are very hard to define, users should have the option to adapt the prompt in correspondence with a health professional until the prompt is appropriately defined.

Finally, an important consideration for the design of a JITAI in the context of KOA is co-designing of the intervention. Mrklas et al [104] describe a frequently occurring problem within the topic of mHealth apps. Users, in this case people with KOA, are usually not involved in the development of the JITAI-app. That can lead to interventions that are not a good fit for the target population. For better individualization, it is crucial to include the opinions and experiences of those affected. This can help to positively influence the user engagement, which has an impact on the effectiveness of a JITAI, as highlighted by previous research [105-107]. The framework by Choi et al [103] addresses this issue and could be used for a higher user-friendliness and thus a higher engagement in the design of JITAIs.

### Limitations and Strengths

Strengths of this review include the detailed search, which has a high number of results within the 4 databases. Furthermore, the chance of finding all relevant articles was increased by examining useful articles for further studies, and thus we were able to include 45 relevant papers. The examination of multiple outcome variables relevant for patients with KOA yields a good fit for a special use case while also providing an overview of JITAIs for health behavior change in general because it was not focused on a specific group of participants.

Limitations of this review include that the terminology and structure of a JITAI have not yet been defined based on a common definition, which hinders screening of eligible articles. A uniform framework for future JITAIs would be of great advantage and would increase comparability [28]. Therefore, this review is oriented and structured based on a JITAI framework [27]. In general, most of the included JITAIs have shown a very high heterogeneity, a low number of participants, and include preliminary studies, which makes it difficult to draw inferences. In addition, either no studies or only very few studies were found that refer to the parameters physical function, stiffness, pain, and also QoL, which limits the explanatory power regarding these parameters. In order to make evidence-based statements, further outcome-specific and high-quality JITAI studies are needed. Finally, body weight and the associated load on the joints are decisive factors in KOA [108], which has not been addressed by this review to maintain a concise focus.

### Conclusions

In summary, a variety of JITAIs can be identified that represent interesting and promising results for behavior change in physiological health outcomes. Although a high degree of heterogeneity in the results was found, patterns have been identified that are associated with intervention effectiveness. Using frequent decision points, device-based measurements of tailored variables accompanied by user input, intervention options tailored to user preferences, and simple decision rules showed the most promising results in previous studies. Transferring this knowledge to the use case of KOA can result in a JITAI to break SB, increase PA, and maintain an optimal knee load for the health benefits of patients. Future JITAIs should be oriented toward a uniform structure and terminology, use microrandomized interventions to be able to draw better inferences, integrate new technological aspects such as sensors for the assessment of knee load, and explore optimal dose-response mechanisms and contextual factors that are linked to intervention effectiveness.

## Appendix

Multimedia Appendix 1 Search strategy.

Multimedia Appendix 2 Key facets and duration of the just-in-time adaptive interventions.

Multimedia Appendix 3 Retention, measurements, and results of the interventions.

Multimedia Appendix 4 Theoretical foundation and behavior change techniques.

Multimedia Appendix 5 PRISMA checklist.

## Abbreviations

BCT: behavior change technique
CG: control group
EMA: ecological momentary assessment
IG: intervention group
JITAI: just-in-time adaptive interventions
KOA: knee osteoarthritis
mHealth: mobile health
PA: physical activity
PICOS: Population, Intervention, Comparison, Outcomes, Study
PRISMA: Preferred Reporting Items for Systematic Reviews and Meta-Analyses
QoL: quality of life
SB: sedentary behavior

## Data Availability

All data generated or analyzed during this study are included in this published article (and its supplementary information files).
