# Supplementary material for: Just-in-Time Adaptive Interventions for Behavior Change in Physiological Health Outcomes and the Use Case for Knee Osteoarthritis: Systematic Review
Source: J Med Internet Res. 2024 Sep 27;26:e54119. doi: 10.2196/54119 (PMC11470223; doi:10.2196/54119)
Supplement: Multimedia Appendix 1 [file jmir_v26i1e54119_app1.docx]

The databases were searched from inception to the date of the electronic search (October 26th, 2022, updated search 09.04.2024). The following terms were searched:

1. To relate to the intervention type, the following keywords were used:

“Ecological AND Momentary AND Intervention OR EMI OR JITAI OR Just-in-Time OR adaptive OR sensor triggered OR real time intervention* OR dynamic tailor* OR real time tailor*”

1. The area of telemedicine and mobile health was covered as follows: "Telemedicine"[Mesh] OR "mobile applications"[Mesh] OR web-based OR mhealth OR mobile health”
2. Finally, restrictions were made regarding the outcomes and a reference was made to physical activity:

“"Locomotion"[Mesh] OR pain OR quality of life OR "movement"[Mesh] OR "exercise"[Mesh] OR physical activity OR physical function OR stiffness”

All categories were linked with an “AND”-Operator. If a database did not allow mesh terms, the associated naming has been removed. In addition, the terms as well as the results and duplicates for all data bases are available in table 1.

Table 1 Literature search and databases

| **Database (date of search)** | **Searchterm** | **Results (duplicates removed)** |
| --- | --- | --- |
| **1.) Pubmed** (26.10.22)  **1.1) Update** (09.04.2024) | **((((Ecological AND Momentary AND Intervention) OR EMI OR JITAI OR Just-in-Time OR adaptive OR sensor triggered OR real time intervention* OR dynamic tailor* OR real time tailor*) AND ("Telemedicine"[Mesh] OR "mobile applications"[Mesh] OR web-based OR mhealth OR mobile health) AND ("Locomotion"[Mesh] OR pain OR quality of life OR "movement"[Mesh] OR "exercise"[Mesh] OR physical activity OR physical function OR stiffness))) *ALL FIELDS***  1.1) AND (("2022/10/22"[Date - Entry] : "3000"[Date - Entry])) | 1,994 results  1.1) 431 results |
| **2.) Scopus** (26.10.22)  **2.1) Update** (09.04.2024) | ((((ecological AND momentary AND intervention) OR emi OR jitai OR just-in-time OR adaptive OR sensor AND triggered OR real AND time AND intervention* OR dynamic AND tailor* OR real AND time AND tailor*) AND (telemedicine OR mobile AND applications OR web-based OR mhealth OR mobile AND health) AND (locomotion OR pain OR quality AND of AND life OR movement OR exercise OR physical AND activity OR physical AND function OR stiffness))) ***ALL FIELDS***  2.1) AND PUBYEAR > 2021 | 712 results *(15 duplicates)*  2.1) 492 results *(12 duplicates)* |
| **3.) Web of Science** (26.10.22)  **3.1) Update** (09.04.2024) | ((((ecological AND momentary AND intervention) OR emi OR jitai OR just-in-time OR adaptive OR sensor AND triggered OR real AND time AND intervention* OR dynamic AND tailor* OR real AND time AND tailor*) AND (telemedicine OR mobile AND applications OR web-based OR mhealth OR mobile AND health) AND (locomotion OR pain OR quality AND of AND life OR movement OR exercise OR physical AND activity OR physical AND function OR stiffness))) ***ALL FIELDS***  3.1) 2022-10-22 | 1,040 results  *(288 duplicates)*  3.1) 200 results *(87 duplicates)* |
| **4.) EBSCO** (26.10.22)  **4.1) Update** (09.04.2024) | ((((Ecological AND Momentary AND Intervention) OR EMI OR JITAI OR Just-in-Time OR adaptive OR sensor triggered OR (real AND time AND intervention*) OR (dynamic AND tailor*) OR (real AND time AND tailor*)) AND ((Telemedicine OR mobile applications OR web-based OR mhealth OR (mobile AND health)) AND ((Locomotion OR pain OR (quality AND of AND life) OR movement OR exercise OR (physical AND activity) OR physical AND function) OR stiffness)))) ***ALL FIELDS***  4.1) 2022 October | 594 results *(268 duplicates)*  4.1) 92 results *(46 duplicates)* |
|  | | **Total: 4340 + 1215 = 5545 results** |
