# Supplementary material for: Just-in-Time Adaptive Interventions for Behavior Change in Physiological Health Outcomes and the Use Case for Knee Osteoarthritis: Systematic Review
Source: J Med Internet Res. 2024 Sep 27;26:e54119. doi: 10.2196/54119 (PMC11470223; doi:10.2196/54119)
Supplement: Multimedia Appendix 2 [file jmir_v26i1e54119_app2.docx]

Table 2: Key facets and duration of the JITAI (IG = intervention group, CG = control group, PA = physical activity, SB =sedentary behavior, EMA = ecological momentary assessment, MET = metabolic equivalent of task, MVPA = moderate to vigorous physical activity)

| **Author (year)** | **Decision points**  Time when intervention decision is made | **Tailoring variables**  Assessment determining intervention delivery (only the JITAI relevant ones) | **Intervention Options**  Possible actions to be performed at decision points | **Decision rules**  Link between intervention options and tailoring variables at decision points | **Duration of intervention** |
| --- | --- | --- | --- | --- | --- |
| **Allicock et al.** [36] | EMAs: (3 types)  1) Daily diary assessments: Each morning (i.e. 30 min after typical wake up).  2) Random sampling assessments: Twice per day.  3) Event sampling assessments: Before and after exercise and meals. | Answers to EMA (related to  PA, not specified).  Self-report.  Not further specified. | Tailored feedback to EMAs.  Not further specified. | Opportune moment:  after participants filled out an EMA  🡪 they received tailored feedback.  Not further specified. | 4 weeks. |
| **Baumann et al.** [37] | Not clearly specified, short time intervals (e.g. minute). | Heart rate and physical activity device-based measured by ECG sensors and accelerometry | Prompt to interrupt inactivity with randomly selected pre-defined motivational messages was sent. | Opportune moment:  during wake time (operationalized based on the heart rate measurement) if 30 minutes of inactivity were detected by the accelerometer.  🡪 prompt was sent | 8 weeks. |
| **Bond et al.** [38]**; Thomas & Bond** [39] | Each minute. | SB (≤1.5 METs).  Device-based measured real-time data by smartphone accelerometry. | Audible prompt to take a physically active break from sedentary behavior was sent.  Participants could respond to the prompt by performing a physical activity break, silencing the prompt, or delaying the prompt by 30 minutes.  Reminders to meet the prompt. | Opportune moments:  1) 30 continuous sedentary minutes (3min walking break).  2) 60 continuous sedentary minutes (6min walking break).  3) 120 continuous sedentary minutes (12min walking break).  was met  🡪 a respective walking break prompt was sent.  If participants performed a physically active break, they received positive feedback plus a green "go" light on the dashboard.  When not silenced/delayed, an additional prompt was sent after 5- and 10-min continued SB | 3 weeks (1 week each condition in counterbalanced order). |
| **Bort-Roig et al.** [40] | Each minute. | SB (sitting time).  Device-based measured real-time data by W@W-App and smartphone inbuilt accelerometer. | Vibration prompt to take a break from sedentary behavior | Opportune moment:  participants sat for more than 60 minutes  a vibration prompt was sent through the mobile phone. | 13 weeks in total.  8 weeks ramping phase & 4 weeks maintenance phase. |
| **Brakenridge et al.** [41,42] | Not clearly specified, short time intervals (e.g. minute). | SB (sitting time), and Posture (pelvic tilt angle).  Device-based measured real-time data by LUMOback activity tracker + mobile app. | Push notification (vibration of sensor). | Opportune moment:  when in a poor lumbar posture + after user-defined time sitting (15min, 30min, 40min, 1h or 2h)  🡪 a vibration was sent if they were in a poor lumbar posture + push notification after sitting. If they selected a time interval for the push notification, then a push notification was sent to the app after the user-defined time interval. | 3 months. With possibility to expand to 12 months. |
| **Carlozzi et al.** [43]  **Wang et al.** [44] | Each minute within a participant chosen 5-hour window. | Step count, sleep quality and HRQOL.  Device-based measured real-time data by accelerometer (Fitbit) and self-reported in the mobile app (CareQOL). | Daily personalized push notification. Notification content was randomly drawn from a pool of over 400 messages of the following types: 1) Data feedback, 2) Facts, 3) Tips, 4) Support. Messages were personalized based on step count and HRQOL data. | Opportune moment:  50/50 chance of receiving notifications each day.  Based on accelerometry data and after daily real-time rating (3 questions) of HRQOL  --> Push notification was sent.  Notifications are triggered in a personalized 5 hour timeframe | **Carlozzi et al.**  2 hour baseline virtual study visit.  10 day run-in period.  90 day intervention  **Wang et al**.  3 months. |
| **Compernolle et al.** [45] | Each minute. | SB (sitting time).  Device-based measured real-time data by accelerometer (Activator). | Vibration of the accelerometer. Participants were instructed to break up their SB each time they received the vibration. | Opportune moment:  after 30 uninterrupted minutes of sitting time  🡪 Vibration prompt was sent | 3 weeks. |
| **Conroy et al.** [46] | Not clearly specified, short time intervals (e.g. minute) within a participant chosen 10-hour window. | PA (stepping time)  Device-based measured real-time data by wrist worn accelerometer. | Prompts were either aiming on self-monitoring or behavioral feedback.  Self-monitoring: Participants had to report the number of minutes being active in the past 2 hours.  Behavioral feedback: Participants get shown the number of minutes being active in the past 2 hours. | Opportune moment:  0-6 prompts per day were randomly sent within a self-selected messaging window and were based on activity duration of last 2 hours. The number, type, and timing of prompts each day were determined randomly by the server without any involvement by the researcher. | 90 days. |
| **Ding et al.** [47] | Not clearly specified, short time intervals (e.g. minute). | Smartphone usage (app-usage api), PA (activity recognition api), SB (SB recognition api), and meals (smart watch).  Device-based measured real-time data by smartphone, Pebble smartwatch Android. | Motivational prompt to walk and prompt to walk more (when already walking). With an explanation of why the prompt occurred. | Opportune moment:  1) Participants overused their smartphone  2) Participants had been sedentary for a long time  3) Participants were walking  4) Participants just had their meals  🡪 Motivational messages tailored to the respective condition    Examples:  SB “You sit too long. Walk for a while"  Smartphone usage “Less Apps. Stand up and Walk" | 3 weeks. |
| **Direito et al.** [48] | Upon opening the app (daily). | PA and SB clustered to "Couch potato", "Potterer", and "Techno-Active".  Device-based measured daily average by Art of Living app. | Behavior change content on the app.  Encouraged daily-life activities, running errands, PA at work, leisure time PA, and replacing SB with light PA. | Opportune moment:  opening the app for the first time at a specific day  🡪 Users were classified into the three activity profiles based on the previous day. Behavior change content was tailored to the respective activity profile. | 8 weeks. |
| **Fiedler et al.** [49] | Each minute. | PA.  Device-based measured real-time data via accelerometer connected to SMART*FAMILY*2.0-app on the study smartphone. | Prompt to break inactivity with a question about the reason for previous inactivity. | Opportune moment:  When inactive for 60 minutes (i.e. <100 steps and <2min with >2MET), during participant specific waketime (max. 7 am-10 pm) if 50 of 60 minutes recorded sensor values were present, and if participants have done less than 60 min MVPA on that day  🡪 prompt was sent out | 3 weeks. |
| **Finkelstein et al.** [50] | Every 10-15 minutes. | PA (steps), Fitbit sync data (website).  Device-based measured real-time data by Fitbit connected to monitoring website. | Prompt to take a break by tailored text message with information about consequences of prolonged time sitting and suggestions of ways to have an activity break. | Opportune moment:  if Fitbit synchronized recently, and if steps <15 in the past hour. No messages were sent during blackout conditions:  1) Self-reported preferences.  2) participant texted S(X) (no messages for the next X hours)  3) participant texted ‘okay’ which meant no messages were sent during the next hour.  🡪 prompt was sent and tailored to the time of day. | 4 weeks. |
| **Freene et al.** [51] | Daily. | PA (steps + time spent active), social opportunity (number of new places visited and time spent there), and variety (how much the individual’s day differs from an average day).  Device-based measured real-time data by Vire App (GPS) and Fitbit Flex (activity data). | Individualized, context-specific, and Data-Driven microbehavioral alternatives (Do´s) for changing a habit and learning new behaviors. | Opportune moment:  If more than 60% of the total available data was available, and participants showed a low score in PA, social opportunity and variety on 3 consecutive days  🡪 an individualized, context specific Data-Driven Do was sent. | 6 weeks. |
| **Fundoiano-Hershcovitz et al.** [52] | Not clearly specified, short time intervals (e.g. minute). | Slouching posture. User defined  sensitivity for posture indication.  Device-based measured real-time data by triaxial accelerometer (UpRight by DarioHealth) located on the upper back. | Vibration cycle with two vibrations and a break of 10s until a good body posture is achieved. | Opportune moment:  When a slouching posture based on user-selected sensitivity, is detected by the sensor. | 8 weeks. |
| **Garland et al.** [53] | Not clearly specified, short time intervals (e.g. minute). | Physiological stress.  Devic-based measured real-time data by photoplethysmogram and pulse oximeter sensor (mEMA app + Garmin Vivosmart smartwatch) | Mindfulness practice when a physiological stress metric obtained. | Opportune moment:   When stress metric exceeded 1 SD of participant´s moving average value  🡪 prompt was sent. | 90 days. |
| **Golbus et al.** [54] | **Activity notification**:  4 time points (morning, lunchtime, midafternoon, evening)  **Exercise planning notification**:  Each evening | Weather, day of week, time of day, and duration within the study.  Mobile study application | Contextually tailored text messages.  Either activity notifications or exercise planning notifications.  Possible personalization with a participant’s preferred name, loss- or gain-framing, and inclusion of an emoji or hyperlink to the study dashboard | Opportune moment:  25% probability of receiving an activity prompt at each decision point. 50% probability of  receiving an exercise message each decision point.  Activity notifications are tailored based on 4 dimensions of contexts: weather, day of week, time of day, and phase of cardiac rehabilitation.  Exercise planning notifications are tailored based on 2 dimensions of contexts: season and phase of cardiac rehabilitation. | 6 months. |
| **Hermens et al.** [55]**; Tabak et al.** [56] | Not clearly specified, short time intervals (e.g. minute). | PA levels (IMA units), previous motivational cues and ‘relevant context factors’.  Device-based measured real-time data by HTC Desire S (smartphone) and activity sensor (ProMove 3D wireless activity tracker, Inertia Technology B.V.) | Motivational message, prompt to walk. Messages could be encouraging, discouraging or neutral. | Opportune moment:  Constant evaluation of the context of the user by machine learning on the app to find suitable situations for delivery of motivational coaching (predicted by analyzing previous cues and learning when a patient was likely to respond well to the message by relating relevant context factors to patient compliance and content). First phase (no previous data), k-nearest neighbor classifier, second phase uses real-time prediction and self-learning by Support Vector Machine implementation (re-trained periodically).  🡪 prompts were sent out and messages were tailored to PA level of participants. | 3 months. Participants were asked to use the app at least four days per week. |
| **Hietbrink et al.** [57] | Twice a day and weekly. | PA (mean daily step count, self-reported activities). Duration of intervention use, type of chronic disease, time of day, type of behavior goal, goal achievement, and the identified barrier toward goal achievement.  Device-based measured real-time data by accelerometer (Fitbit Charge 2) and self-reported activities (E-Supporter app) | Tailored motivational messages selected from a pool of 425 messages.  Feedback based on goal achievement and reflective questions for goal achievement of the upcoming week.  Step goal which gradually became more difficult. Psychological exercises when a goal was not sufficiently reached and motivation, self-efficacy, mood, stress, or planning was the identified barrier for goal achievement | Opportune moment:  Daily motivational message and feedback based on goal achievement. Intervention options were tailored based on if-then rules to the variables of behavior goal, phase of behavior change, type of chronic disease, time of day, and goal achievement. There were three types of decision rules: (1) rules that triggered the type of motivational message, (2) rules that triggered feedback on goal achievement, and (3) rules that triggered a type of psychological exercise. | 9 weeks. |
| **Hiremath et al.** [58] | Each minute. | Wheelchair-based PA (MVPA).  Device-based measured real-time data by Android-based smartphone, wrist-worn smartwatch, and Bluetooth-based wheel rotation monitor (Pano-Bike).  App: Personal Health Informatics and Rehabilitation Engineering (PHIRE) | Congratulatory Messages and near-real-time, personalized feedback through the smartphone (audio and/or vibration) and smartwatch (vibration). | Opportune moment:  about (≥3min) of MVPA (or higher) PA was performed  🡪 Near-real-time feedback  + Congratulatory message  + if goal attained congratulation on the goal attainment (then back to ^1^)  + if goal exceeded feedback about total number of MVPA that day (then back to ^1^)  + if goal not reached total minutes of MVPA needed for goal attainment displayed (then back to ^1^)  + if neither of previous conditions are met no additional message is sent (unclear what this includes then back to ^1^) | 3 months.  1st month - baseline.  2^nd^ month – PA feedback.  3th month – PA feedback with JITAI. |
| **Ismail & Al Thani** [59] | Each minute. | SB (≤67 steps/40 min), location, weather information, time information.  Device-based measured real-time data by smartphone, MotiFit (CG = MotiFit Lite) app + accelerometer, Open Weather Application Programming Interface (API). | CG: Static reminder messages.  IG: Context-aware motivational messages.  Context aware messages could relate to goal achievement, weather, nearby restaurants, parks, gyms, and malls (total of 260 unique motivational messages). | Opportune moment:  If one of the following conditions was  - 30 min before lunchbreak  - 1 hour before end of the working day  - sitting for 40 minutes.  🡪 a motivational message was sent  IG: the motivational message was tailored to the context using a decision tree (see Multimedia Appendix 3 of the original publication) | 66 days. |
| **Klasnja et al.** [60] | Up to 5 actionable suggestions a day sent at times the participants thought they would have an opportunity for PA. | PA (steps), weather (not specified).  Device-based measured real-time data by Fitbit tools (Fitbit Charge 2 wrist-worn activity tracker + Fitbit App). | Actionable suggestions to walk or disrupt SB. 50% walking suggestions, and 50% anti-sedentary suggestions, if prolonged inactive.  Library of over 500 text messages. | Opportune moment:  at the predefined 5 decision points  🡪 suggestions were micro-randomized:  - 0.7 no suggestion  - 0.15 walking suggestion  - 0.15 anti-sedentary and tailored to time of day, day of the week, and current weather | 16 weeks. |
| **Li et al.** [61] | Not clearly specified, short time intervals (e.g. minute). | SB (< 100 counts/min).  Device-based real-time data by Actiwatch 2 Smartwatch (Moto 360) and Android tablet.  Apps: Elderfit. | Prompt that encourages participants toward their goals. | Opportune moment:  If >90 mins of inactivity were detected during the day  🡪 sedentary alert was sent to the smartwatch | 6 weeks in total.  1 week pretest.  4 weeks intervention.  1 week posttest. |
| **Low et al.**[62] | Not clearly specified, short time intervals (e.g. minute) | PA (steps), severity of 10 symptoms (e.g. pain, fatigue (scale 0-10)).  Device-based real-time data by Fitbit Versa smartwatch + Google Pixel 2 Smartphone + Detective Activity to Supporting Health (DASH) app on smartphone + smartwatch. | Activity prompt “Ready for a short walk?”.  Possible responses on  either the watch or the phone: Yes, No, or Snooze. | Opportune moment:  Based on severity of symptoms a threshold for SB triggers was set for 2 cases:   - All symptoms were rated less than 7 then 60 min SB (less than 50 steps)  -Any symptom was rated 7 or higher then 120 min SB  🡪 prompt was sent  If participants chose Snooze the prompt was repeated 15 minutes later. If No then they were asked to indicate reasons. Positive feedback message was sent in all cases if 30 or more steps were logged within 15  minutes of an activity prompt. | **Low et al. (49)**  From a minimum of 2 weeks before surgery to 30 days after discharge  Average: 66 days (47-81) |
| **Low et al.** [63] | See  Low et al. [62] | See  Low et al. [62] | See  Low et al. [62] | See  Low et al. [62] | From a mean of 20 days before surgery to 30 days after discharge  Average: 57 days (44-92) |
| **Martin et al.** [64] | 3 times per day:  morning (wake time),  mid-day (lunch time),  evening (leisure time). | Online questionnaire pre-study for personalizing text messages (self-report on 16 personal and clinical characteristic).   PA (steps).   Device-based real-time data by Fitbug Orb (accelerometer) and Fitbug-compatible smartphone. | Positive reinforcement or booster messages. | Opportune moment:  At each decision point, smart texts were customized to the participant, depending on if a participant was  on track 🡪 positive reinforcement  or not 🡪 booster message to attain their daily goal (10.000 steps). | 5 weeks:  Run-in (1 week).  Phase 1 (weeks 2 to 3).  Phase 2  (weeks 4 to 5). |
| **McEntee et al.** [65] | Daily. | PA (MVPA)  Device-based measured by a wrist-worn accelerometer (ActiGraph). | Tailored feedback messages, performance feedback with reinforcement, and new exercise goals. | Prompt messages were selected in a random sequence without replication from the pool of messages and delivered daily to each individual. Adaptive goals were based on a percentile-rank algorithm using the 60^th^ percentile of the moving average of the previous days. Differential feedback texts related to their goal achievement (e.g., “You're on target! Goal met! 10 min today… Goal for 4/1 is 8 min.”). | 12 months. |
| **Nurmi et al.** [66] | morning notification (new content), 5 pm notification (goal progress), not clearly specified (further behavior change tools) | Step count, heart rate. Device-based measured by a wrist-worn activity bracelet that synchronized with the app in 10-minute intervals (Xiaomi Mi Band; Firstbeat Technologies (heart rate)). | Prompts to increase motivation and provide self-regulation techniques. E.g. prompts when the user reached a goal. Further options not clearly specified. | Opportune moment:  When users reached a goal, a tailored prompt is provided. When a certain pre-defined time of the day is reached tailored reminders and interactive prompts are sent out. | 40-day trial including 12 three-day active study periods, 2 additional control days, and a  2-day lead-out period in which all app features were available |
| **Pellegrini et al.** [67] | Not clearly specified, short time intervals (e.g. minute). | SB (<100 counts/min).  Device-based measured real-time data by a wireless accelerometer.  Smartphone (Android). | Prompt to take a break from sedentary position by noise or vibration (participants preference). Question about adherence to the prompt with options:  - Stand  - Extend  - Cannot stand  - Ignore | Opportune moment:  more than 20 min SB were assessed by the accelerometer  🡪 a reminder prompt was triggered encouraging the participant to stand up for at least two minutes.  If participants stated they wanted to stand but did not, additional reminders were sent every 2 min. | 4 weeks. |
| **Rabbi et al.** [68] | Not clearly specified, short time intervals (e.g. minute). | PA (min spent in certain activity), SB (min spent stationary), location.  🡪 Continuous life-log which clusters previous activities using machine-learning model (Gaussian Mixture Model).  Device-based measured real-time data by Smartphone accelerometer and GPS, Android. Self-reported PA if no device-based measurement was possible. | Suggestions for physical activities based on user's frequent past behavior + occasional higher-calorie-burning activities. | Opportune moment:  based on automated sensing (accelerometer and GPS) when participants were in specific locations (on the way to work) or sedentary for prolonged period  🡪 Prompts were sent out Daily 10 messages relating to 90% frequent activities, 10% infrequent behaviors based on multi-armed bandit were sent. | 3 weeks. |
| **Rabbi et al.** [69] | See Rabbi et al. ​(52)​. | See Rabbi et al. ​(52)​. Additionally, user input. | See Rabbi et al. ​(52)​. | See Rabbi et al. ​(52)​.  Additionally, user preferences for the suggestions were included to allow for change in circumstances. | 9 weeks (delivery ranged between 7 and 9 weeks). |
| **Rabbi et al.** [70] | Not clearly specified, short time intervals (e.g. minute). | Behavioral data and PA.  🡪 grouped behavior routines using machine-learning model (Gaussian Mixture Model).  Device-based measured real-time data by Smartphone-inbuilt accelerometer and GPS. Self-reported input if no device-based measurement was possible.  MyBehaviorCBP-App. | MyBehaviorCBP-generated recommendations based on their participants behaviors. E.g. “Walk for 8 more minutes near Thompson St”; “Take 3-minute walking breaks every hour”. Maximum PA of suggestions is 60 minutes. | Opportune moment:  based on automated sensing (accelerometer and GPS) when participants were in specific locations (on the way to work) or sedentary for prolonged period. The app then uses a sequential decision-making algorithm (multi-armed bandit) to select and rank recommendations that are maximized to be both actionable and beneficial.  🡪 Prompts were sent out Prioritization of behaviors:  - Most frequent and repeated  - less intensive  - small changes  Suggestions also use contextual information (e.g. road names)  80% of the recommendations are easy and have been frequently followed before. 20% for exploration of other recommendations | 5 weeks.  1^st^ week – baseline  2^nd^-3^rd^ week- random suggestions  4^th^-5^th^ week -intervention. |
| **Radhakrishnan et al.** [71] | Not clearly specified, real-time, short time intervals (e.g. minute). | Weight monitoring (kg) and PA (steps).  Device-based measured real time data by Withing Go activity tracker, Withing Body smart weighting scale, Withing Health Mate app, and the Heart Health Mountain SCDG (Sensor-Controlled Digital Game). | Game alerts, avatar´s health status, messages, and incentives. | Opportune moment:  not further specified  🡪 The SCDG (Sensor-Controlled Digital Game) was tailored to participant's real-time heart failure self-management | 12 weeks. |
| **Robertson et al.** [35] | Twice per hour. | PA (steps).  Device-based measured real-time data by Fitbit Alta devices synchronized to Fitabase.  Steps2Health app. | 54 total messaging blocks designed to target autonomous regulation, autonomy, relatedness, and competence. Those included hyperlinks to various resources for healthy living. | Opportune moment:  if certain number of steps was reached  🡪 prompt was sent including motivational messages, playful (digital) experience of destination, hyperlinks to videos for muscle strengthening, stress-reduction techniques, and positive interaction with a fictive character. | Until they reach 166.000 steps on their Fitbit devices. |
| **Sporrel et al.** [72] | Every hour from 8 am to 8 pm -  Location based. | PA (frequency,  duration, speed, and distance), location (GPS/ Beacons). time of the day, the day of the week, weather, and agenda availability.  Device-based measured real-time data by Playful Active Urban Living (PAUL)   - Basic PAUL (IG 1) - Smart PAUL (IG 2)   Smart Paul app can optimize the timing of reminders with a self-learning module. | Reminder messages based on a reinforcement learning algorithm.  Location-based strength exercise prompts. | Opportune moment:  Pre-learned reinforcement learning model up to a max of 14 prompts per week  🡪 prompts are sent including motivational suggestions and information on progress toward their goal or on performing PA. Additionally, location-based (predetermined) strength exercise prompts containing instruction videos on the exercise were sent out (e.g. push-up in the park). | 4 weeks. |
| **Stuber et al.** [73] | Not clearly specified, real-time, short time intervals (e.g. minute). | Step count, GPS data, BCT preferences measured at baseline  Device-based measured real-time data by mobile coach app and step counter app (IG = both; CG = step counter app only) | 2-6 messages per day on 3 different topics:  1) feedback tailored to step count level 2) contextually tailored prompts near walking locations 3) behavior change advice | Opportune moment:  Step count message is sent once per day tailored to the user's performance level.  Contextual tailored messages are sent whenever the users are near preselected green spaces (max one every 4 hours).  2 messages are sent tailored to BCT preferences | 12 months. |
| **Tabak et al.** [74,75] | Every two hours. | PA (counts per minute).  Device-based measured real-time data by three-dimensional-accelerometer (MTx-W sensor, Xsens Technologies, Enschede, The Netherlands) and a smartphone (HTC P3600/3700). | (1) a short summary of activity behavior and (2) advice on how to improve or maintain the activity behavior.  - encouraging cues (>10% deviation below reference line)  - discouraging cues (>10% deviation above reference line)  - neutral cues (≤10% deviation with reference line) | Opportune moment:  every 2 hours  🡪 prompts were sent and tailored to the difference between the measured activity and the reference line at the moment the cue was generated. | 4 weeks, first week baseline measurement to establish reference line |
| **Valle et al.** [76,77] | Not clearly specified, real-time, short time intervals (e.g. minute). | Individual-level PA data.  Device-based measured via accelerometer (ActiGraph). | motivational messages, prompts to engage in PA, reinforcement or praise, questions about PA | 3 prompts per week are sent at random times within 3 timeframes (9 am-1 pm, 1-5 pm, 5-9 pm) | 6 months. |
| **Van Dantzig et al.** [78] | Each minute. | **Study 1:** computer activity (keyboard strokes  and mouse movement).  Device-based measured real-time data by own smartphone, Activity monitor, and software installed on computer.  **Study 2:** SB (when the position of the 3 coordinates did not change exceeding 0.3 over 5 seconds), Device-based measured real-time data by accelerometer of iPhone 3G. | Study 1:  Prompt (vibration + buzzing sound) to take a break from computer activity.  Study 2:  SB with a persuasive message recommending PA.  Messages from a pool of 32:  - 8 Authority messages  - 8 Scarcity messages  - 9 Consensus messages  - 7 Commitment messages | **Study 1**: Opportune moment:  whenever 30 mins of nearly uninterrupted computer activity was recorded  🡪 a short SMS containing a hyperlink was sent to the participant’s smartphone, when clicked they were shown a message persuading them to be more active. Randomly selected from the pool. Max 3 messages a day with min 2 hours in between them  **Study 2**: Opportune moment:  Participants could choose time intervals, default was set to 60 min SB  🡪 prompt to take a break of 5 min, with a general daily activity goal of 50 min. | 1 day (study 1).  6 weeks (study 2). |
| **Van Dantzig et al.** [79] | Not clearly specified, real-time, short time intervals (e.g. minute). | PA (steps), time of day,  location (from Bluetooth-enabled iBeacons), weather (free API of the OpenWeatherMap service), and behavioral events (participants achieved a step target or set a new step record). Device-based measured by Smartphone, wrist-worn activity tracker, Philips health watch, operating system not reported. | Prompt with audio cue. Suggestions for PA from over 500 message templates.  Feedback about number of steps tailored to specific contexts. | Opportune moment:  during actionable moments in personally relevant geofence zones (e.g., home, work, nature area).  🡪 prompts based on Time events, Location events, and Behavior events. Prompts were only sent if certain conditions were met (e.g., the message would only be sent between 9AM and 12AM, in rainy weather, or at a specific location). | 2 calibration weeks, 1 week of intervention, and 1 week fade-out. |
