# Supplementary material for: Just-in-Time Adaptive Interventions for Behavior Change in Physiological Health Outcomes and the Use Case for Knee Osteoarthritis: Systematic Review
Source: J Med Internet Res. 2024 Sep 27;26:e54119. doi: 10.2196/54119 (PMC11470223; doi:10.2196/54119)
Supplement: Multimedia Appendix 3 [file jmir_v26i1e54119_app3.docx]

Table 3: Retention, measurements and results of the interventions (IG = intervention group, CG = control group, PA = physical activity, SB =sedentary behavior, MVPA = moderate to vigorous physical activity, QoL = quality of life, LIPA =light-intensity physical activity; ↑ = significant improvement, ↓ = significant deterioration (if not otherwise described))

| **Author (year)** | **Retention** | **measure used, how measured, follow-up period** | **Significant over time or vs. group differences at p<.05 (significant/total outcomes)** |
| --- | --- | --- | --- |
| **Allicock et al.** [36] | All participants (n = 22) completed the study.  100%. | Mean daily MVPA, daily minutes of MVPA, low/high light intensity, sedentary time, mean daily sedentary time.  PA and SB: Behavioral Risk Factor Surveillance System (BRFSS) physical activity questionnaire and accelerometer (Worn 7 consecutive days).  Baseline, 4 weeks (end of intervention) and 8 weeks follow-up) | **Over time:**  ***PA (1/16):***  Daily minutes of MVPA ↓ CG 10.95 (SD = 9.93) min (baseline – 4 weeks)  ***SB (0/8)***  **Vs. control:**  ***PA (0/8)***  ***SB (0/4)*** |
| **Baumann et al.** [37] | 170 out of 643 completed the study.  26%. | Steps per day, daily minutes of MVPA, disruption of sedentary behavior, daily minutes of inactivity.  Follow-up 8 weeks post-intervention. | **Vs. control:**  ***PA (1/2):***  MVPA min/day ↑ between the 2 individualized (JITAI) IGs and the other IGs + CG from pre- to post-intervention.  ***SB (1/2):***  Disruption counts/day ↑ between the 2 individualized (JITAI) IGs and the other IGs + CG from pre- to post-intervention. |
| **Bond et al.** [38]**; Thomas & Bond** [39] | 30 out of 35 completed the study.  86%. | ***Bond et al. (2014):***  **Primary outcome:** Change in daily time spent in sedentary behavior.  **Secondary outcomes:** Light and moderate-to-vigorous PA.  Device-based measured through independent measurement (separate accelerometer).  No follow up.  ***Thomas & Bond (2014):***  Daily number of walking prompts, walking breaks, and daily minutes of walking breaks  Device-based measured through onboard accelerometer of smartphone.  No follow-up. | ***Bond et al. (2014):***  **Over time:**  ***PA (6/6):***  Time spent in light and MVPA ↑ in all 3 physical activity break conditions  ***SB (3/3):***  ↓ in all 3 physical activity break conditions  **Vs. group:**  ***PA (1/6):***  Light intensity activity ↑ 3-min condition vs 12-min condition  ***SB (1/3):***  ↓ 3-min condition vs 12-min condition    ***Thomas & Bond (2015):***  **Over time:**  ***PA (3/3):***  Number of daily minutes spent in walking breaks ↓ for all conditions  **Vs. group:**  ***PA (2/3):***  Minutes spent in walking breaks ↑ 3- and 6-min condition vs 12 min condition |
| **Bort-Roig et al.** [40] | 42 out of 90 in IG.  22 out of 51 in CG.  45% total (IG: 47%; CG: 43%) | Stepping time (hours), MVPA (min), LIPA (hours), standing time (hours), sedentary time (hours), total sedentary bouts (number; <5 min, 5–10 min, 10–20 min, <20 min, >20 min, 20–30 min, 30–40 min, <40 min, >40 min, 40–60 min, >60 min, and >90 min), total sedentary time (minutes; same durations). Additionally, total time spent in light intensity and moderate-to-vigorous PA was determined by using previously validated count-to-activity thresholds.  Follow-up after 12 weeks of intervention. | **Over time:**  ***PA (4/20):***  MVPA (total/weekend) ↑, Stepping (work) ↑, Occupational light intensity activity ↑  ***SB (6/125):*** Sedentary breaks (non-working time 20-30min and weekend 5-10min) ↑, shorter sedentary bouts ↑  **Vs. control:**  ***PA (0/20)***  ***SB (3/125):*** Number of daily breaks and time spent on short sedentary bouts (5-10/ <20 min; weekend) ↑ |
| **Brakenridge et al.** [41,42] | IG: 66 baseline – 61 received intervention – 41/37 (overall/work) at 3 months – 23/21 (overall/work) at 12 months  CG: 87 baseline – 86 received intervention – 68/65 (overall/work) at 3 months – 38/36 (overall/work) at 12 months    2018 IG intervention use:  Frequent user (n = 9; 16 – 48 days)  Infrequent user (n = 16; 5 – 15 days)  Limited user (n = 13; 1 - 4 days)  Nonuser (n = 24; 0 days) | **2016**:  **Primary outcomes:** Average time per day spent sitting during work hours and overall.  **Secondary outcomes:** Stress, physical health QoL, mental health QoL, the average time per day spent in prolonged sitting bouts (sitting time accrued in continuous bouts of 30 min or more), standing, and stepping; the number of steps per day; and the average time period between sitting bouts.  PA and SB were measured objectively through ActivPAL3 activity monitor and QoL was measured subjectively via questionnaire.  Follow-up after 3 and 12 months.    **2018:**  Time per 10 hours at work and time per 16 hours awake spent engaged in sitting, prolonged sitting (≥30 min continuously), nonprolonged sitting (<30 min continuously), standing, and stepping.  Sitting and activity data were collected at baseline, 3 months, and 12 months (24/7).  Objectively measured through activ3PAL activity monitor.  Follow-up after 3 and 12 months. | **2016:**  **Over time (3 months):**  ***PA (1/12):***  CG: Standing, min/16h ↑ (14.6 min)  ***SB (0/12)***  ***QoL (0/6)***  **Over time (12 months):**  ***PA (5/12):***  CG/IG: Standing, min/10 h ↑ (CG: 39.2 min; IG: 27.4 min), Standing, min/16 h ↑ (CG: 33.5 min; IG: 26.9 min)  IG: Stepping, min/10 h ↑ (9,1 min)  ***SB (8/12):***  CG/IG: Sitting, min/10 h ↓ (CG: 40.5 min; IG: 35.5 min), Prolonged sitting, min/10 h ↓ (CG: 41.3 min; IG: 45.7 min), Sitting, min/16 h ↓ (CG: 32.1 min; IG: 35 min),  CG: Time between sitting bouts ↑ (1.7 min), Prolonged sitting, min/16 h ↓ (30 min)  ***QoL (0/6)***  **Vs. control (3months):**  ***PA (0/6)***  ***SB (0/6)***  **Vs. control (12 months):**  ***PA (2/6):***  Stepping, min/16 h ↑ (20.6 min), Number of steps/16 h ↑ (846.5 steps)  ***SB (0/6)***    **2018:**  **Over time:**  **Usage for at least 5 days:**  ***PA (0/4)***  ***SB (4/6):***  Nonprolonged sitting time (work) ↑ after removal of 2 influential cases n.s. prolonged sitting time (work) ↓  Prolonged sitting time (wake) ↓  Nonprolonged sitting time (wake) n.s. but ↑ after removal of 1 influential case  **Time frame activity was measured:**  ***PA (0/4)***  ***SB (2/6):***  Nonprolonged sitting time (wake) ↑ Prolonged sitting time (wake) n.s. but ↓ after removal of 1 influential case |
| **Carlozzi et al.** [43]  **Wang et al.** [44] | **Carlozzi et al.:**  69 out of 70 completed the study.  99 %.  **Wang et al.:**  36 out of 36 completed the study.  100 %. | HRQOL scores of caregiver strain, anxiety, and depression as a  computer adaptive test throughout the 3 month home monitoring period. The final HRQOL scores for each CAT event were used in the analysis, weekly step count (not used for effectiveness).  Self-reported through questionnaire within CareQOL-App.  No follow-up. | **Carlozzi et al.**  **Over time:**  ***QoL (2/20)***  IG/CG Anxiety over time ↓ (better)  **vs. control:**  ***QoL (3/10):***  Caregiver Strain, Depression, and Sleep-Related Impairment ↓ (better) for IG compared to CG    **Wang et al.**  **HRQOL (1/9)**  Caregiver strain ↓ for high JITAI frequency vs no JITAI |
| **Compernolle et al.** [45] | 26 out of 26 completed the study.  100 %. | Break in sedentary behavior (within 1, 3, and 5 minutes) after feedback.  Timely categorized: 6-9AM, 9AM-noon, noon-3PM, 3-6PM, 6-9 PM, 9PM-midnight.  Device-based measured through Activator.  No follow-up. | Out of 2628 vibrations, 379 (14,4 %; 1min), 570 (21,7%; 3min), and 798 (30,4 %; 5min) resulted in SB-breaks.  **Over time (real-time):**  ***SB (2/15):***  **3-minute:**  Noon-3PM ↑ vs 6-9AM  **5-minute:**  Noon-3PM ↑ vs 6-9AM |
| **Conroy et al.** [46] | 58 out of 58 completed the study.  100 %. | PA (Daily step count and MVPA).  Device-based measured through Actigraph and Fitbit.  No follow-up. | **Within persons:**  ***PA (3/6)***  Step count and MVPA ↑ from pre-post  Step count ↑ associated with number of self-monitoring prompts |
| **Ding et al.** [47] | 16 out of 19 completed the study.  84 %. | Step counts during weeks 2–4 and perceived effectiveness of the app in terms of encouraging the participant to walk more.  Device-based measured through in-built sensor/accelerometer (smartphone or smartwatch).  Exit interview after four weeks, some data were collected at 10 pm each day (e.g. whether app encouraged them to do other activities).  No follow-up. | **Over time:**  ***PA (4/4)***  Step counts for IG and CG ↑  Self-reported effectiveness of the app to encourage them to do other physical activities for IG and CG↑  **Vs. control:**  ***PA (1/2)***   Self-reported effectiveness of the app to encourage them to do other physical activities ↑ |
| **Direito et al.** [48] | 62 out of 69 completed the study.  90% | **Secondary Outcomes**: Daily time spent in total-, light-, and moderate to vigorous-intensity PA, and SB.  PA and SB were device-based measured via the Art of Living app and the PA determinants were self-reported by validated instruments.  Follow-up survey after 8 weeks. | **Over time:**  ***PA (1/3):***  Time spent in light PA ↑ (2.2min/day)  ***SB (0/1)*** |
| **Fiedler et al.** [49] | 80 out of 98 completed the study.  82 %. | MET and step count.  Device-based measured through 3-axial accelerometer and smartphone.  Follow-up questionnaire 4 weeks after the post measurement. | **Within-person effects (step count):**  ***PA (3/3)***  Within 60/90/120 minutes answered ↑  **Between-person effects (step count):**  ***PA (0/3)***  **Within-person effects (MET):**  ***PA (1/3)***  Within 60minutes answered ↑  **Between-person effects (MET):**  ***PA (0/3)*** |
| **Finkelstein et al.** [50] | 27 out of 30 participants completed the study.  90 %. | **Primary outcome:** Number of episodes of prolonged inactivity (> 2 h) per day, but only when the inactivity reminder was active.  **Secondary outcome:** Step count.  Device-based measured through in-built sensor / accelerometer.  Eight weeks, of which four weeks with the inactivity monitor active and four weeks with the monitor inactive.  No follow-up. | **Over time:**  ***PA (0/3)***  ***SB (2/3):***  Two-hour slots with less than 20 steps (inactivity) during “message-on” period ↓  Inactivity Group A between periods (within-group difference) message-on vs off ↓ |
| **Freene et al.** [51] | 19 out of 20 patients completed the 6-week follow-up and 12 the 16-week follow-up. For missing data at the follow-up, the last value was brought forward. Therefore, all 20 participants could be analyzed.  100 %. | **Primary outcome:** SB (min/day), percentage of SB per day, duration of SB bouts per day, number of SB bouts per day, number of SB breaks per day.  **Secondary outcome:** MVPA, light PA, vector magnitude (VM), steps per day, exercise capacity (6-min walk test, 6MWT), blood pressure, quality of life (MacNew Heart Disease Health-Related Quality of Life Questionnaire, MacNew).  Device-based measured through accelerometer (ActiGraph, ActiSleep) and GPS (Vire app).  Follow-up after 6 weeks (end of intervention) and 16 weeks. Participants had still access to the vire app after 6 weeks, but didn´t receive any Do´s.  Follow-up after 6- and 16-weeks. | **Over time (6weeks):**  ***PA (1/5)***  6-minute walking-test ↑  ***SB (0/5):***  ***QoL (4/4):***  ↑ in all domains  **QoL:**  ↑ over time in all domains  ***Physical Function (1/2):***  Systolic blood pressure ↓  **Over time (16 weeks):**  ***PA (1/5):***  6-minute walking-test ↑  ***SB(0/5)*** *(no significance testing due to small sample size)*  Over time ↓ (Cohen´s d = 0,54)  Percentage of day spent in SB ↓ **(**Cohen´s d = 0.25)  ***QoL (4/4):***  ↑ in all domains  ***Physical Function (1/2):***  Systolic blood pressure ↑ |
| **Fundoiano-Hershcovitz et al.** [52] | 981 users out of 6.098 using the app were included for the main analysis based on inclusion criteria.  14%. | Pain level and subjective posture using the numerical rating scale (NRS) from 0-10 (0, no pain – mostly slouched; 10, extreme pain – mostly upright). For all three parameters a mean value over a 7-days interval was calculated.  No follow-up. | **Over time:**  ***SB (1/2)***  subjective posture quality ↑ first 4 weeks and maintained afterwards  ***Pain (1/2)***  Pain level ↓ (better) first 4 weeks and maintained afterwards  **Additional:**  Association between training duration and pain |
| **Garland et al.** [53] | 63 out of 66 completed the study.  95 %. | Pain level.  Self-reported near-real time data by EMAs.  No follow-up. | **Over time:**  **Pain (2/2)**  pain level ↓ (better) for IG and ↑ (worse) for CG  **Vs. control**:  ***Pain (1/1***)  Pain level ↓ (better) compared to control group over time |
| **Golbus et al.** [54] | 214 out of 223 completed the study.  96 %. | **Primary outcome:**  Change in 6-min walk distance (6 months; Fitbit & Apple Watch)  **Secondary outcome:**  Change in 6-min walk distance (3 months; Fitbit & Apple Watch)  Change in mean daily step count (6 months; Fitbit & Apple Watch)  **Exploratory outcomes:**  Change in mean daily step count (3 months; Fitbit & Apple Watch)  Change in EuroQol Visual Analoque Scale  No follow-up | **Vs. control:**  ***PA (1/8)***  6 min walking distance (Fitbit) ↑ for IG vs CG from baseline to 3 months assessment  ***QoL (0/1)*** |
| **Hermens et al.** [55]**; Tabak et al.** [56] | 8 out of 10 patients completed the study.  80 %. | Activity level, activity balance (%), subjective activity, exercise capacity, and health status.  Device-based measured through independent measurement  (separate accelerometer) and 6-min walking test to measure exercise capacity.  During the three months of intervention and follow-up for one week at three months after the intervention. | *(No significance testing since data were reported for each participant)*  **Over time (end of intervention):**  **PA (12/32)**  5 participants ↑ activity level  4 participants ↑ activity balance  3 participants ↑exercise capacity  ***QoL (5/8):***  5 participants ↑ health status  **Over time (3-month follow-up):**  **PA (10/32)**  3 participants ↑ activity level  4 participants ↑ activity balance  3 participants ↑exercise capacity  ***QoL (5/8):***  5 participants ↑ health status    The percentage of days on which goals were achieved ranged between 23 to 59% for activity levels and between 21 and 85% for balance. |
| **Hietbrink et al.** [57] | 17 out of 20 completed the study.  85 %. | Mean daily step count.  Device-based measured through accelerometer (Fitbit Versa 2) | **Over time:**  ***PA (1/2)***  baseline to postintervention ↑ median daily step count |
| **Hiremath et al.** [58] | 16 out of 20 completed the study.  80 %. | Leisure Time Physical Activity Questionnaire for people with SCI (LTPAQ-SCI), and Wheelchair User’s Shoulder  Pain Index (WUSPI).  Light-, moderate- and vigorous intensity PA.  Energy expenditure (kcal) and travelled distance (miles).  Smartphone, smartwatch, and wheel rotation monitor.  No follow-up. | *No significance testing due to small sample size*  ***PA feedback (2/3):***  4 out of 16 (light-intensity) and 3 out of 16 (moderate-intensity) PA ↑ (>+10%)  ***PA feedback with JITAI (2/3):***  6 out 19 (light-intensity) and 9 out 16 (moderate-intensity) PA ↑ (>+10%).  3 out of 16 (light-intensity) and 7 out of 16 (moderate-intensity) PA **↓** (<- 10%) |
| **Ismail & Al Thani** [59] | 58 out of 58 participants completed the study.  100 %. | PA through International Physical Activity Questionnaire (IPAQ) pre-study.  SB (if step count/hour < 67) and daily active time.  Device-based measured through smartphone inbuilt accelerometer.  No follow-up. | **Over time:**  ***SB (1/2):***  MotiFit: Breaking inactivity ↑  ***PA (0/2)***  **Vs. control:**  ***PA (0/1)*** |
| **Klasnja et al.** [60] | 45 out of 51 participants provided baseline data (88 %).  42 started the intervention (82 %).  29 provided data for follow-up (57 %). | **Primary outcome:** Average steps per day at baseline and at the end of the study.  **Secondary outcome**: Steps, sitting time, standing time, and sit-to-stand transitions.  Steps measured by Fitbit at weeks 1–2, weeks 15–16.  Average daily step count, measured by Fitbit, over the 16-week intervention period.  Pre–post comparison of activPAL step counts.  Step count, measured by Fitbit, in the 30 min following each randomization.  Follow-up after 16 weeks. | **Over time:**  ***PA (2/4):***  Daily step count ↑ 1,866 steps  Stepping time ↑ 21 min/day  ***SB (0/1)*** |
| **Li et al.** [61] | 8 out of 8 completed the study.  100 %. | **Secondary outcomes:**  PA: mean level of PA.  SB: duration of sedentary activity (% of waking time), and sedentary time.  The Physical Activity Scale for the Elderly (PASE).  Device-based measured by wrist worn Actiwatch 2.  1 week follow-up after the 4-week intervention. | **Over time:**  ***PA (2/3):***  PA ↑ at posttest (41.5 counts/min)  Self-reported PA ↑ at posttest (96.2 PASE Score)  ***SB (4/4):***  Sedentary time ↓ during intervention (-42.3 min) and ↓ posttest (-87.4 min)  Sedentary activities (waking time) ↓ during intervention (-5.7%) and posttest (-8%) |
| **Low et al.**[62] | 14 out of 15 participants completed the study.  93 %. | Daily step count and average SB bout duration.  Device-based measured with Fitbit Versa smartwatch.  No follow-up. | **Over time:**  ***PA (1/3):***  Step counts ↓ from preoperative to inpatient recovery  ***SB (1/3):***  SB bouts ↑ from preoperative to inpatient recovery |
| **Low et al.** [63] | 23 out of 26 completed the study.  88 %. | QoL, daily step count and average SB bout duration.  Device-based measured with Fitbit Versa smartwatch and QoL via questionnaire.  No follow-up. | **Over time:**  ***PA (1/2):***  Step counts ↓ from preoperative to inpatient recovery  ***SB (1/2):***  SB bouts ↑ from preoperative to inpatient recovery  ***QoL (3/6):***  QoL ↓ from preoperative to inpatient recovery  depressive symptoms ↑ (worse) from preoperative to inpatient recovery  physical symptoms ↑ (worse) from preoperative to inpatient recovery  **vs control:**  ***PA (0/1)***  ***SB (1/1):***  maximum overall SB bouts ↑ in the intervention compared to control group.  ***QoL (0/3)*** |
| **Martin et al.** [64] | 47 out of 48 participants completed the study.  98 %. | PA through International Physical Activity Questionnaire (IPAQ) pre-study.  **Primary outcomes:** Mean change in daily step count and attainment of the step goal.  **Secondary outcome:** Changes in daily activity time and aerobic time.  Device-based measured through Fitbug Orb accelerometer.  No follow-up. | **Over time:**  **Phase 1**  ***PA (0/3)***  **Phase 2**  ***PA (3/3):***  Daily steps (37% increase) ↑  Total activity time ↑ (21 min/day)  Aerobic time ↑ (13 min/day)  **Vs. no texts:**  ***PA (3/3):***  Daily steps ↑ (+2534 steps/day)  Aerobic time ↑  Total activity time ↑  **Vs. blind:**  ***PA (3/3):***  Daily steps ↑ (+3376 steps/day)  Aerobic time ↑  Total activity time ↑ |
| **McEntee et al.** [65] | 512 out of 512 completed the study.  100 %.  128.993/157.285 (82%) accelerometer observations. | Likelihood of any MVPA, Daily MVPA bout minutes.  Device-based measured using a wrist-worn accelerometer.  No follow-up. | **Over time:**  **PA (2/2)**  likelihood of any MVPA ↑ in all intervention groups  Daily MVPA bout minutes ↑ in all intervention groups  **Vs. control:**  **PA (1/2)**  likelihood of any MVPA from baseline to intervention adaptive goals ↑ relative to static goals. |
| **Nurmi et al.** [66] | 15 out of 15 completed the study.  100 %.  A total of 88.3% (530/600) of  usable data points were recorded. | Daily steps.  Device-based measured using a wrist-worn accelerometer.  No follow-up. | **Within-person vs. control condition**  **PA (0/2)** |
| **Pellegrini et al.** [67] | 8 out of 9 completed the intervention.  89 %. | Proportion of the day spent sedentary, proportion of the day spent in light PA, and MVPA, breaks/day in sedentary time, break duration, break intensity.  Device-based measured through independent measurement (separate accelerometer).  One month, which was during the intervention period. | **Over time:**  **PA (1/3)**  Light PA ↑  **SB (3/3)**  Sedentary time ↓  Breaks/day in sedentary time ↑  Break duration ↓  **Within-person (light PA):**  **PA (7/8)**  7/8 participants ↑ time spent in light physical activity. |
| **Rabbi et al.** [68] | 17 out of 17 completed the 3-week period.  100 %. | Walking trends and walking time.  Device-based measured by smartphone-inbuilt GPS.  No follow-up. | **Vs. control:**  ***PA (2/2):***  Walking trends ↑ (IG: 7 out of 9)  Walking time ↑ (IG: +10 min) |
| **Rabbi et al.** [69] | 16 out of 16 completed the study.  100 %. | Behavior change (calorie loss in exercise) from participants’ logs of daily activity.  Self-report.  Minutes of walking per day.  Device-based measured by smartphone-inbuilt GPS.  No follow-up. | **Vs. control:**  ***PA (2/2):***  Minutes of walking per day ↑  Calories burned through non-walking exercises per day ↑ |
| **Rabbi et al.** [70] | 10 out of 10 completed the study.  100 %. | Number of minutes spent walking per day, number of minutes spent in nonwalking exercises per day.  Device-based measured with smartphone inbuilt sensors.  Pain level from 0 to 10.  Self-reported in daily evening survey.  No follow-up. | **Vs. control:**  ***PA (1/2):***  Daily walked minutes ↑  ***Pain (0/1)*** |
| **Radhakrishnan et al.** [71] | 30 out of 38 participants attended the 12-week surveys (79 %) and 27 remained in the 24-weeks survey (71 %). | Cumulative steps for each day and average steps over 6 and 12 weeks, heart failure-related functional status, and QoL.  PA behavior was device-based measured through Withings Go sensor.   Data about functional status and QoL were obtained with the Kansas City Cardiomyopathy Questionnaire (KCCQ).  Surveys were completed at 6, 12 and 24 weeks (follow-up) after baseline survey. | **Over time:**  ***PA (1/2):*** *no significance testing*  Steps: Modest ↑ IG; Modest ↓ CG 6^th^ to 12^th^ week  ***QoL (5/6):***  ↑ (baseline to 6/12/24 weeks) in both groups except baseline to 6 weeks for IG  ***Physical Function (2/6):***  HF functional status ↑ after 6 weeks in IG  HF functional status ↑ after 24 weeks in CG |
| **Robertson et al.** [35] | 75 out of 78 completed the study.  96 %. | PA pre-study and for MVPA:  Godin Leisure-Time Exercise Questionnaire.  PA: MVPA (self-reported), and step count device-based measured by Fitbit.  PA pre-study and for MVPA:  Godin Leisure-Time Exercise Questionnaire.  28 days follow-up after the intervention (4 weeks). | **Over time:**  ***PA (5/9):***  Self-reported: ↑ group-by-time interaction in favor of IG (52% ↑)  Step count: ↑ group-by-time interaction  ↑ for IG during the intervention  Result extended in the follow-up. |
| **Sporrel et al.** [72] | 20 out of 23 completed the study.  87 %.  9 out of 12 (BasicPaul) were analyzed.  75 %. | MVPA.  Device-based measured through smartphone-inbuilt accelerometer (ActiLife), and pre-posttest PA measures with additional hip-worn accelerometer (ActiGraph GT3X+).  Follow-up questionnaire 1 week after intervention. | **Over time:**  ***PA (0/1)***  **Vs. control:**  ***PA (0/1)*** |
| **Stuber et al.** [73] | 361 out of 421 completed the study.  86 %. | Daily step count.  Device-based measured through smartphone’s built-in pedometer or accelerometer.  No follow-up. | **Vs. control:**  ***PA (0/1)*** |
| **Tabak et al.** [74,75] | 30 out of 34 completed the study.  88 %. | Number of steps per day and health status (2014b).  Activity counts per minute (cpm) (2014c).  Device-based measured through MTx-W 3D accelerometer, Yamax Digiwalker 200 (pedometer) and subjectively through Multidimensional Fatigue Inventory (MFI-20).  No follow-up. | **2014b:**  **Over time:**  ***PA (0/6)***  ***QoL (1/2)***  Health status ↑ IG  **Within-person (1/13 IG; 3/15 CG)**  1 patient (IG) ↓ health status.  3 patients (CG) ↓ health status.  **2014c:**  **Over time:**  ***PA (1/3):***  PA level after corrected for reactivity  ↑ on a group level.  ***PA (4/33): Within-person***  Activity level ↑ |
| **Valle et al.** [76,77] | **Valle et al. (effect):**  251 out of 280 completed the study.  90 %.  **Valle et al. Physical:**  236 out of 280 delivered accelerometer data.  84 %.  246 out of 280 delivered self-reported data.  88 %. | Total PA, MVPA, steps per day, SB.  Device-based measured through wrist worn accelerometer and self-reported data via questionnaires.  12 months follow-up. | **Valle et al. (Effect):**  **Over time:**  **PA (9/14)**  Accelerometer:  total PA IG ↑  MVPA IG and CG ↑  steps IG ↑  Self-report:  total PA IG and CG ↑  MVPA IG and CG ↑  light PA IG ↑  **SB (0/8)**  **Vs. control:**  **PA (0/7)**  after removal of outliers ↑ accelerometer based MVPA in IG vs CG  **SB (0/4)**  **Valle et al. (Physical):**  **Over time:**  **PA (8/14)**  Accelerometer:  baseline-12 months:  MVPA IG and CG ↑  6 months-12 months:  steps IG ↓  Self-report:  baseline-12 months:  total PA IG and CG ↑  MVPA IG and CG ↑  light PA IG ↑  **SB (1/12)**  Accelerometer:  6-12 months:  SB ↑ (worse)  **Vs. control:**  **PA (1/7)**  self-report:  ↑ total PA in IG vs CG baseline to 12 months  **SB (0/3)** |
| **Van Dantzig et al.** [78] | 86 out of 86 completed the study.  100 %. | Computer activity (proxy for sedentary behavior) and physical activity (calories) during the 30 mins before a text message were compared with computer activity and physical activity during 30 mins after the text message.  Device-based measured through independent measurement  (separate accelerometer); computer activity through specially installed software.  No follow-up. | **Study 2:**  **Over time:**  ***PA (1/1):***  Calories ↑  ***SB (1/1):***  Computer activity ↓  **Vs. control:**  ***PA (1/1):***  Calories in IG ↑  ***SB (1/1):***  Computer activity ↓  **Over time x vs. condition:**  ***SB:***  Computer activity IG (10.0 min) ↓ compared to CG (5.9 min) |
| **Van Dantzig et al.** [79] | 60 out of 70 completed the study.  86 %. | Average daily step count.  Device-based measured through independent accelerometer.  Collected during a 2-week calibration period, 1-week intervention period and 1-week fade-out period. | Each group was divided into three clusters:  cluster 1: steps<=6500, cluster 2: 6500 < steps<=9500, and cluster 3: steps> 9500.  **Over time:**  **PA (4/18)**  Cluster 1:  CG coaching ↑ vs calibration and fadeout  IG coaching ↑ calibration.  Cluster 2:  IG coaching ↑ vs calibration and fadeout. |
