# Supplementary material for: Just-in-Time Adaptive Interventions for Behavior Change in Physiological Health Outcomes and the Use Case for Knee Osteoarthritis: Systematic Review
Source: J Med Internet Res. 2024 Sep 27;26:e54119. doi: 10.2196/54119 (PMC11470223; doi:10.2196/54119)
Supplement: Multimedia Appendix 4 [file jmir_v26i1e54119_app4.docx]

| **Author (year of publication)** | **Theory base** | **BCTs included in the intervention** | **BCTs included in the control condition** |
| --- | --- | --- | --- |
| **Allicock et al.** [31] | Social Cognitive Theory Control Theory. | Tailored messages:  2.3 Self-monitoring of behavior  7.1 Prompts/cues  10.2 Material reward (behavior)  Newsletter:  4.1 Instruction on how to perform a behavior  5.1 Information about health consequences  N = 5 | 2.3 Self-monitoring of behavior  10.2 Material reward (behavior)  N = 2 |
| **Baumann et al.** [32] | Not reported. | 2.2 Feedback on behavior  2.3 Self-monitoring of behavior  2.6 Biofeedback  4.1 Instruction on how to perform a behavior  5.1 Information about health consequences  7.1 Prompts/cues  N = 6. | 3 comparisons arms with different BCTs. |
| **Bond et al.** [33]**; Thomas & Bond** [34] | Not reported. | Face to face session:  5.1 Information about health consequences  Intervention:  1.1 Goal setting (behavior)  1.4 Action planning  1.6 Discrepancy between current  behavior and goal  2.2 Feedback on behavior  7.1 Prompts/cues  10.2 Material reward (behavior)  10.4 Social reward  Final face-to-face visit:  2.2 Feedback on behavior  N = 9 | No comparison arm. |
| **Bort-Roig et al.** [35] | Not reported. | 1.1 Goal setting (behavior)  1.6 Discrepancy between current  behavior and goal  2.2 Feedback on behavior  2.3 Self-monitoring of behavior  7.1 Prompts/cues   N = 5 | 1.6 Discrepancy between current  behavior and goal  2.3 Self-monitoring of behavior  N = 2 |
| **Brakenridge et al.** [36,37] | Not reported. | 2.2 Feedback on behavior  2.3 Self-monitoring of behavior  5.1 Information about health consequences  7.1 Prompts/cues   N = 4 | 2.3 Self-monitoring of behavior  4.1 Instruction on how to perform the behavior  5.1 Information about health consequences  N = 3 |
| **Carlozzi et al.** [38]  **Wang et al.** [39] | Behavioral activation theory | 2.2. Feedback on behavior  2.3. Self-monitoring of behavior  4.1 Instruction on how to perform the behavior  7.1 Prompts/cues  10.1 Material incentive (behavior)  11.2 Reduce negative emotions  N = 6 | 2.2. Feedback on behavior  2.3. Self-monitoring of behavior  10.1 Material incentive (behavior)  N = 3 |
| **Compernolle et al.** [40] | Not reported. | 2.2 Feedback on behavior  2.3 Self-monitoring of behavior  7.1 Prompts/cues  N = 3 | No comparison arm. |
| **Conroy et al.** [41] | Not reported. | 1.1 Goal setting (behavior)  1.6 Discrepancy between current behavior and goal  2.2 Feedback on behavior  2.3 Self-monitoring of behavior  7.1 Prompts/cues  N = 5 | No comparison arm. |
| **Ding et al.** [42] | Fogg Behaviour Model, Goal Setting Theory (Locke and Latham), Habit Formation Theory. | 1.1 Goal setting (behavior)  2.2 Feedback on behavior  4.2 Information about antecedents  7.1 Prompts/cues   8.1 Behavioral practice/rehearsal   8.3 Habit formation   N = 6 | 1.1 Goal setting (behavior)  2.2 Feedback on behavior  7.1 Prompts/cues: randomly sent   N = 3 |
| **Direito et al.** [43] | Integrated Behavior Change Model. | TODAY app consisted of 33 BCTs (not all mentioned in the publication):  1.1 Goal setting (behavior)  1.2 Problem solving  1.4 Action planning  1.6 Discrepancy between current behavior and goal  2.2 Feedback on behavior  2.3 Self-monitoring of behavior  3.2 Social support (practical)  3.3 Social support (emotional)  4.1 Instruction on how to perform a behavior  5.1 Information about health consequences  5.2 Salience of consequences  5.3 Information about social and environmental consequences  6.1 Demonstration of the behavior  7.1 Prompts/cues  12.1 Restructuring the physical environment  N = 15 | No comparison arm. |
| **Fiedler et al.** [44] | Self-Determination Theory. | 1.1 Goal setting (behavior)  1.5 Review behavior goal(s)  1.6 Discrepancy between current behavior and goal  2.2 Feedback on behavior  2.3 Self-monitoring of behavior  3.1 Social support (unspecified)  5.1 Information about health consequences  7.1 Prompts/cues  10.2 Material reward (behavior)  10.4 Social reward  13.1 Identification of self as role model  N = 11 | 10.2 Material reward (behavior)   N = 1 |
| **Finkelstein et al.** [45] | Not reported. | 1.4 Action planning  2.2 Feedback on behavior  4.1 Instruction on how to perform the  behavior  7.1 Prompts/cues  N = 4 | 1.4 Action planning  2.2 Feedback on behavior  4.1 Instruction on how to perform the  behavior  7.1 Prompts/cues (but no inactivity reminder)   N = 4 |
| **Freene et al.** [46] | Not reported. | 1.1 Goal setting (behavior)  1.4 Action planning  2.2 Feedback on behavior  3.1 Social support (unspecified)  5.1 Information about health consequences  7.1 Prompts/cues  12.1 Restructuring the physical environment  N = 7 | No comparison arm. |
| **Fundoiano-Hershcovitz et al.** [47] | Not reported. | 1.1 Goal setting (behavior)  1.6 Discrepancy between current behavior and goal  2.2 Feedback on behavior  4.1 Instruction on how to perform the behavior  7.1 Prompts/cues  N = 5 | No comparison arm. |
| **Garland et al.** [48] | Not reported. | 2.2 Feedback on behavior  2.3 Self-monitoring of behavior  3.2 Social support (practical)  4.1 Instruction on how to perform the behavior  7.1 Prompts/cues  N = 5 | 3.2 Social support (practical)  4.1 Instruction on how to perform the behavior  N = 2 |
| **Golbus et al.** [49] | Not clearly specified. | 1.1 Goal setting (behavior)  1.5 Review behavior goal  2.2 Feedback on behavior  2.3 Self-monitoring of behavior  2.4 Self-monitoring of outcomes of behavior  4.1 Instruction on how to perform the behavior  7.1 Prompts/cues  N = 7 | 2.3 Self-monitoring of behavior  2.4 Self-monitoring of outcomes of behavior  N = 2 |
| **Hermens et al.** [50]**; Tabak et al.** [51] | Not reported. | 1.1 Goal setting (behavior)  1.6 Discrepancy between current behavior and goal  2.2 Feedback on behavior  4.1 Instruction on how to perform the behavior  6.2 Social comparison  7.1 Prompts/cues  N = 6 | No comparison arm. |
| **Hietbrink et al.** [52] | Health Action Process Approach, Rothman´s Theory of Maintenance and Marlatt´s Relapse Prevention Theory | 1.1 Goal-setting (behavior)  1.2 Problem-solving  1.4 Action planning  1.5 Review behavior goal  2.2 Feedback on behavior  2.3 Self-monitoring of behavior  2.4 Self-monitoring of outcomes of behavior  3.1 Social support, including motivational interviewing  3.2 Social support (practical)  4.1 Instruction on how to perform the behavior  5.1 Information about health consequences  5.6 Information about emotional consequences  6.3 Information about others’ approval  7.1 Prompts and cues  8.3 Habit formation  9.1 Credible source  9.2 Pros and cons  9.3 Comparative imagining of future outcomes  11.2 Reduce negative emotions  13.2 Framing and reframing  15.1 Verbal persuasion about capability  15.3 Focus on past success  N = 22 | No comparison arm. |
| **Hiremath et al.** [53] | Not reported. | 1.1 Goal setting (behavior)  2.2 Feedback on behavior  2.3 Self-monitoring of behavior  4.1 Instruction on how to perform the behavior  7.1 Prompts/cues  10.4 Social reward  N = 6 | No comparison arm. |
| **Ismail & Al Thani** [54] | Not reported. | 1.1 Goal setting (behavior)  2.2 Feedback on behavior  5.1 Information about health consequences  7.1 Prompts/cues  10.4 Social reward  N = 5 | 1.1 Goal setting (behavior)  2.2 Feedback on behavior  5.1 Information about health consequences  7.1 Prompts/cues (static)  10.4 Social reward   N = 5 |
| **Klasnja et al.** [55] | Not reported. | 1.1 Goal setting (behavior)  2.2 Feedback on behavior  2.3 Self-monitoring of behavior  7.1 Prompts/cues  N = 4 | No comparison arm. |
| **Li et al.** [56] | Self-efficacy Theory. | 1.1 Goal setting (behavior)  2.3 Self-monitoring of behavior  2.7 Feedback on outcome(s) of behavior  7.1 Prompts/cues  10.10 Reward (outcome)  N = 5 | No comparison arm. |
| **Low et al.**[57] | Not reported. | 2.2 Feedback on behavior  2.3 Self-monitoring of behavior  7.1 Prompts/cues  10.4 Social reward  N = 4 | No comparison arm. |
| **Low et al.** [58] | Not reported. | 2.2 Feedback on behavior  2.3 Self-monitoring of behavior  7.1 Prompts/cues  10.4 Social reward  N = 4 | 2.3 Self-monitoring of behavior  N = 1 |
| **Martin et al.** [59] | Not reported. | 1.1 Goal setting (behavior)  2.2 Feedback on behavior  2.3 Self-monitoring of behavior  7.1 Prompts/cues  10.4 Social reward  N = 5 | 1.1 Goal setting  N = 1 |
| **McEntee et al.** [60] | Not clearly specified. | 1.1 Goal setting (behavior)  1.6 Discrepancy between current behavior and goal  1.7 Review outcome goal(s)  2.2 Feedback on behavior  4.1 Instruction on how to perform the behavior  7.1 Prompts/cues  10.2 Material reward (behavior)  10.4 Social reward  N = 8 | 1.1 Goal setting (behavior)  1.6 Discrepancy between current behavior and goal  1.7 Review outcome goal(s)  2.2 Feedback on behavior  4.1 Instruction on how to perform the behavior  7.1 Prompts/cues  10.2 Material reward (behavior)  10.4 Social reward  N = 8 |
| **Nurmi et al.** [61] | Self-Determination Theory | 1.1 Behavioral goal setting  1.3 Outcome goal setting  1.4 Action planning  1.5 Review behavior goal(s)  1.6 Discrepancy between current behavior and goal  1.7 Review outcome goals  2.2 Feedback on behavior  2.3 Self-monitoring of behavior  2.4 Self-monitoring of  outcome(s) of behavior  2.6 Biofeedback  2.7. Feedback on outcome(s)  of behavior  10.4 Social reward  15.2 Mental rehearsal of successful performance  15.3 Focus on past success  Additional BCT: Linking behavioral goals with outcome goals  N = 15 | 1.1 Behavioral goal setting  1.4 Action planning  1.5 Review behavior goal(s)  1.6 Discrepancy between current behavior and goal  2.2 Feedback on behavior  2.3 Self-monitoring of behavior  10.4 Social reward  N = 7 |
| **Pellegrini et al.** [62] | Not reported. | 2.2 Feedback on behavior  7.1 Prompts/cues  N = 2 | No comparison group. |
| **Rabbi et al.** [63] | Learning theory, Social Cognitive Theory, Fogg’s Behaviour Model. | 1.1 Goal setting (behavior)  1.4 Action planning  2.2 Feedback on behavior  2.3 Self-monitoring of behavior  2.5 Monitoring of outcome(s) of behavior without feedback  7.1 Prompts/cues  8.1 Behavioral practice/rehearsal  8.3 Habit formation  8.7 Graded tasks  10.2 Reconstructing the physical environment   N = 10 | 1.1 Goal setting (behavior)  1.4 Action planning   N = 2 |
| **Rabbi et al.** [64] | Decision-making theory models: multi-armed bandit and paretofrontier.   Fogg’s Behaviour Model, Social Cognitive Theory. | Version 2.0 included all BCTs of version 1.0 (see above).  Additional BCT in version 2.0:  1.5 Review behavior goal(s)  10.2 Material reward (behavior)   N = 12 | Version 2.0 included all BCTs of version 1.0 (see above).  Additional BCT in version 2.0:  10.2 Material reward (behavior)   N = 3 |
| **Rabbi et al.** [65] | Not reported. | 1.4 Action planning  2.2 Feedback on behavior  2.3 Self-monitoring of behavior  4.1 Instruction on how to perform the behavior  7.1 Prompts/cues  8.7 Graded tasks  N = 6 | No comparison arm. |
| **Radhakrishnan et al.** [66] | Fogg´s Behavioral Model. | 1.1 Goal setting (behavior)  2.2 Feedback on behavior  5.1 Information about health consequences  7.1 Prompts/cues  10.4 Social reward  10.8 Incentive (outcome)  N = 6 | 5.1 Information about health consequences  N = 1 |
| **Robertson et al.** [30] | Self-Determination Theory. | 1.1 Goal setting (behavior)  2.2 Feedback on behavior  2.3 Self-monitoring of behavior  4.1 Instruction on how to perform the behavior  6.1 Demonstrating of the behavior  7.1 Prompts/cues  N = 6 | 1.1 Goal setting (behavior)  2.2 Feedback on behavior  2.3 Self-monitoring of behavior  N = 3 |
| **Sporrel et al.** [67] | Fogg´s Behavioral Model and Capability, Opportunity and Motivation Behavior Model. | 1.1 Goal setting (behavior)  1.5 Review behavior goal(s)  2.2 Feedback on behavior  2.3 Self-monitoring of behavior  2.4 Self-monitoring of outcome(s) of behavior  4.1 Instruction on how to perform a behavior  6.1 Demonstration of the behavior  7.1 Prompts/cues  8.7 Graded tasks  10.2 Material reward (outcome)  10.4 Social reward  N = 11 | 1.1 Goal setting (behavior)  1.5 Review behavior goal(s)  2.2 Feedback on behavior  2.3 Self-monitoring of behavior  2.4 Self-monitoring of outcome(s) of behavior  4.1 Instruction on how to perform a behavior  6.1 Demonstration of the behavior  7.1 Prompts/cues (static)  8.7 Graded tasks  10.2 Material reward (outcome)  10.4 Social reward   N = 11 |
| **Stuber et al.** [68] | Social cognitive theory | 1.1 Goal setting (behavior)  1.4 Action planning  2.3 Self-monitoring of behavior  3.1 Social support (unspecified)  3.3 Social support (emotional)  4.1 Instruction on how to perform the behavior  5.1 Information about health consequences  5.6 Information about emotional consequences  6.2 Social comparison  6.3 Information about others´ approval  7.1 Prompts/cues  10.3 Non-specific reward  N = 12 | 2.3 Self-monitoring of behavior  N = 1. |
| **Tabak et al.** [69,70] | Not reported. | 1.1 Goal setting (behavior)  1.6 Discrepancy between current behavior and goal  2.2 Feedback on behavior  4.1 Instruction on how to perform the behavior  6.2 Social comparison  7.1 Prompts/cues  N = 6 | No comparison arm. |
| **Valle et al.** [71,72] | Social  cognitive theory | 1.1 Goal setting (behavior)  1.2 Problem solving  1.3 Goal setting (outcome)  1.4 Action planning  1.5 Review behavior goal(s)  1.6 Discrepancy between current behavior and goal  1.7 Review outcome goal(s)  2.2 Feedback on behavior  2.3 Self-monitoring of behavior  2.4 Self-monitoring of outcome(s) of behavior  2.7 Feedback on outcome(s) of behavior  3.1 Social support (unspecified)  3.2 Social support (practical)  3.3 Social support (emotional)  4.1 Instruction on how to perform a behavior  5.1 Information about health consequences  5.4 Monitoring of emotional consequences  5.3 Information about social and environmental consequences  5.4 Monitoring of emotional consequences  5.6 Information about emotional consequences  6.1 Demonstration of the behavior  6.2 Social comparison  7.1 Prompts/cues  8.1 Behavioral practice/rehearsal  8.2 Behavior substitution  8.3 Habit formation  8.4 Habit reversal  9.1 Credible source  9.2 Pros and Cons  10.1 Material incentive (behavior)  10.2 Material reward (behavior)  10.4 Social reward  10.7 Self-incentive  10.9 Self-reward  10.10 Reward (outcome)  11.2 Reduce negative emotions  12.1 Restructuring the physical environment  12.2 Restructuring the social environment  13.1 Identification of self as role model  13.2 Framing/reframing  13.4 Valued self-identity  13.5 Identity associated with changed behavior  15.1 Verbal persuasion about capability  15.2 Mental rehearsal of successful performance  15.3 Focus on past success  15.4 Self-talk  16.2 Imaginary Reward  N = 47 | 1.1 Goal setting (behavior)  1.3 Goal setting (outcome)  1.4 Action planning  1.6 Discrepancy between current behavior and goal  2.2 Feedback on behavior  2.3 Self-monitoring of behavior  2.4 Self-monitoring of outcome(s) of behavior  2.7 Feedback on outcome(s) of behavior  3.1 Social support (unspecified)  3.2 Social support (practical)  3.3 Social support (emotional)  4.1 Instruction on how to perform a behavior  5.1 Information about health consequences  5.6 Information about emotional consequences  6.1 Demonstration of the behavior  6.2 Social comparison  7.1 Prompts/cues  8.1 Behavioral practice/rehearsal  8.2 Behavior substitution  8.4 Habit reversal  9.1 Credible source  10.3 Non-specific reward  10.4 Social reward  10.10 Reward (outcome)  11.2 Reduce negative emotions  12.1 Restructuring the physical environment  12.5 Adding objects to the environment  N = 27 |
| **Van Dantzig et al.** [73] | Social influence strategies defined by Cialdini. | 1.1 Goal setting (behavior)  1.4 Action planning  1.9 Commitment  2.2 Feedback on behavior  2.3 Self-monitoring of behavior  5.1 Information about health consequences  6.2 Social comparison  7.1 Prompts/cues  9.1 Credible source  N = 8 | Study 1:   No comparison arm.   Study 2:    2.3 Self-monitoring of behavior    N = 1 |
| **Van Dantzig et al.** [74] | Not reported. | 1.1 Goal setting (behavior)  1.4 Action planning  2.2 Feedback on behavior  4.1 Instruction on how to perform the behavior  7.1 Prompts/cues  8.1 Behavioral practice/rehearsal  10.4 Social reward  N = 7 | 1.1 Goal setting (behavior)  1.4 Action planning  2.2 Feedback on behavior  4.1 Instruction on how to perform the behavior   7.1 Prompts/cues (static)   N = 5 |
